# Spatial mapping of the total transcriptome by in situ polyadenylation

**DOI:** 10.1038/s41587-022-01517-6

**Published:** 2022-11-03

**Authors:** David W. McKellar, Madhav Mantri, Meleana M. Hinchman, John S. L. Parker, Praveen Sethupathy, Benjamin D. Cosgrove, Iwijn De Vlaminck

**Affiliations:** 1 Meinig School of Biomedical Engineering, Cornell University, Ithaca, NY USA; 2 Department of Computational Biology, Cornell University, Ithaca, NY USA; 3 Baker Institute for Animal Health, College of Veterinary Medicine, Cornell University, Ithaca, NY USA; 4 Department of Biomedical Sciences, College of Veterinary Medicine, Cornell University, Ithaca, NY USA

**Keywords:** Transcriptomics, Viral host response, Long non-coding RNAs, miRNAs, Small RNAs

## Abstract

Spatial transcriptomics reveals the spatial context of gene expression, but current methods are limited to assaying polyadenylated (A-tailed) RNA transcripts. Here we demonstrate that enzymatic in situ polyadenylation of RNA enables detection of the full spectrum of RNAs, expanding the scope of sequencing-based spatial transcriptomics to the total transcriptome. We demonstrate that our spatial total RNA-sequencing (STRS) approach captures coding RNAs, noncoding RNAs and viral RNAs. We apply STRS to study skeletal muscle regeneration and viral-induced myocarditis. Our analyses reveal the spatial patterns of noncoding RNA expression with near-cellular resolution, identify spatially defined expression of noncoding transcripts in skeletal muscle regeneration and highlight host transcriptional responses associated with local viral RNA abundance. STRS requires adding only one step to the widely used Visium spatial total RNA-sequencing protocol from 10x Genomics, and thus could be easily adopted to enable new insights into spatial gene regulation and biology.

## Abstract (web summary)

Spatial RNA sequencing is extended beyond poly-A transcripts to capture the full transcriptome.

## Main

Spatial transcriptomics provide insight into the spatial context of gene expression^1–5^. Current methods are restricted to capturing polyadenylated transcripts and are not sensitive to many species of non-A-tailed RNAs, including microRNAs, newly transcribed RNAs and many nonhost RNAs. Extending the scope of spatial transcriptomics to the total transcriptome would enable observation of spatial distributions of regulatory RNAs and their targets, link nonhost RNAs and host transcriptional responses, and deepen our understanding of spatial biology.

Recent single-cell RNA-sequencing methods, Smart-Seq-Total^6^ and VASA-seq^7^, have adapted enzymatic polyadenylation to enable plate-based and microfluidic-based single-cell total RNA-sequencing, respectively. These methods demonstrated that non-A-tailed RNAs comprise information on cell type and cell state, but both methods lack spatial information. Here, we demonstrate spatial total RNA-sequencing (STRS), a method that enables spatial profiling of both the A-tailed and non-A-tailed transcriptome. This is achieved with a simple modification of a commercially available protocol for spatial RNA-sequencing (Visium, 10x Genomics). STRS uses poly(A) polymerase to add poly(A) tails to RNAs in situ. STRS otherwise follows conventional protocols to capture, spatially barcode and sequence RNAs. STRS is compatible with existing approaches for sequencing-based spatial transcriptomics, is straightforward to implement and adds minimal cost and time to an already widely used commercially available workflow. STRS enables the capture of many RNAs that are missed by conventional workflows, including noncoding RNAs, newly transcribed RNAs and viral RNAs. To demonstrate the versatility of the method, we applied STRS to study the regeneration of skeletal muscle after injury and the pathogenesis of viral-induced myocarditis.

## Results

### STRS enables capture of coding and noncoding RNAs

**Fig. 1:**
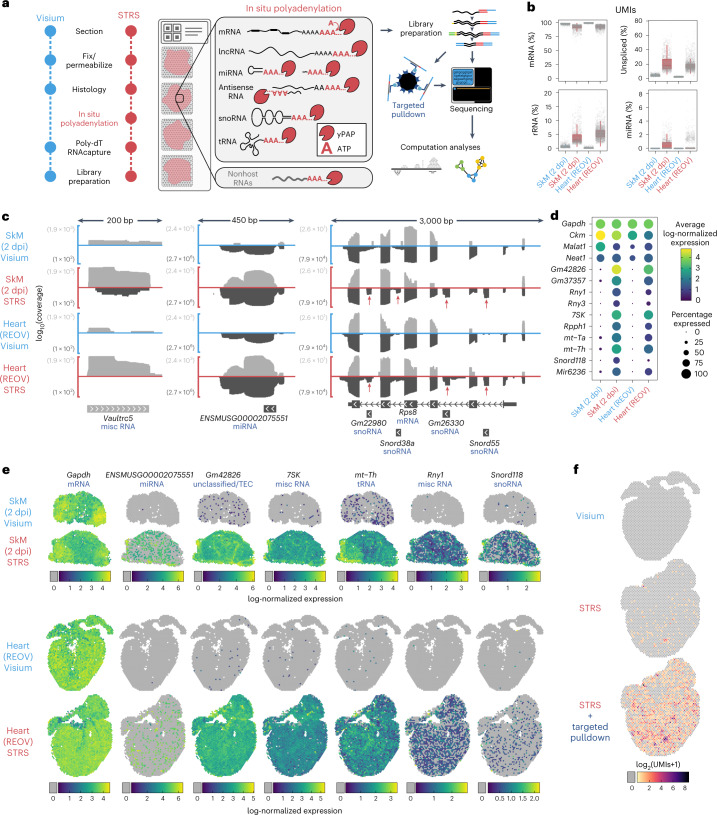
In situ polyadenylation enables spatial profiling of noncoding and nonhost RNAs. **a**, Workflow for STRS. **b**, Comparison of select RNA biotypes between Visium and STRS datasets. The *y* axis shows the percent of UMIs for each spot. The box shows median and quartile values, and whiskers show 1.5 times interquartile range. **c**, Detection of coding and noncoding RNAs between Visium and STRS workflows. Color scale shows average log-normalized UMI counts. Dot size shows the percentage of spots in which each RNA was detected. **d**, Log_10_-transformed coverage of deduplicated reads mapping to sense (light gray) and antisense (dark gray) strands at the Vaultrc5, ENSMUSG00002075551 and Rps8 loci. Annotations shown are from GENCODE M28 and include one of the five isoforms for Rps8 as well as the four intragenic features within introns of Rps8. **e**, Spatial maps of coding and noncoding transcripts for Visium and STRS workflows. Spots in which the transcript was not detected are shown as gray. Color scale indicates log-normalized expression. **f**, Detection of REOV transcripts using the standard workflow, STRS and STRS with targeted pulldown enrichment. Spots in which the virus was not detected are shown as gray.

STRS adds a single step to a commercially available method for spatial RNA-sequencing (Visium Spatial Gene Expression, 10x Genomics) to capture the total transcriptome^8^. As in the Visium method, the sample is first sectioned, fixed with methanol and stained for histology. After imaging, the sample is rehydrated and then incubated with yeast poly(A) polymerase for 25 min at 37 °C. This enzyme adds poly(A) tails to the 3′ end of all RNAs so that endogenous poly(A) tails are extended, and non-A-tailed transcripts are polyadenylated. After in situ polyadenylation, STRS again follows the Visium protocol without modification (Fig. 1a). One important feature of the Visium method that we leverage in STRS, is its use of a strand-aware library preparation. We found that strandedness is critical for the study of noncoding and antisense RNAs (see below) and must be considered in bioinformatic analyses (Supplementary Fig. 1).

To test the performance and versatility of STRS, we applied it to two distinct mouse tissue types: injured hindlimb muscle^5^ and virally infected heart tissue^4^. We quantified the percentage of unique molecules (UMIs) as a function of RNA biotype (GENCODE M28 annotations; Fig. 1b). Compared with the Visium method, we found similar counts for protein-coding and other endogenously polyadenylated transcripts (Supplementary Figs. 1–3). STRS enabled robust detection of several types of noncoding RNAs that are poorly recovered or not detected at all by the Visium method, including ribosomal RNAs (rRNAs; mean of 5.4% and 2.6% of UMIs for STRS and Visium respectively; computed across all Visium and STRS samples included in this study), microRNAs (miRNAs; 0.4% in STRS versus 0.004% in Visium), transfer RNAs (tRNAs; 0.4% in STRS versus 0.02% in Visium), small nucleolar RNAs (snoRNAs; 0.2% in STRS versus 0.002% in Visium), and several other biotypes (Fig. 1b and Supplementary Figs. 2–6). STRS libraries also had an increased fraction of unspliced transcripts (2.7% in Visium versus 18.3% in STRS). Unspliced or nascent RNA counts have been used to predict transcriptional trajectories for single cells. Improved detection of nascent RNAs may enable more accurate trajectory imputation and reveal the dynamics of spatial gene expression. Finally, STRS libraries had an increased fraction of reads that map to intergenic regions, reflecting increased capture of unannotated transcriptional products (22.2% in STRS versus 9.5% in Visium; Supplementary Fig. 1b,c).

We also compared RNA biotype profiles of STRS to existing single-cell total RNA-sequencing datasets (Smart-Seq-Total^6^ and VASA-drop^7^) and found that STRS performed similarly to VASA-drop, but Smart-Seq-Total had a higher fraction of non-protein-coding RNAs (Supplementary Fig. 6). We further tested the compatibility of in situ polyadenylation with single-nucleus RNA-sequencing and found that these data were even more enriched with intergenic reads (6.8% versus 40%; Supplementary Fig. 7c). Given the enrichment of intergenic reads, we applied TAR-scRNA-seq^9^, a gene-annotation-free pipeline that identifies transcriptionally active regions in single-cell RNA-sequencing data. We found that in situ polyadenylation enables around three times higher capture of UMIs mapping to transcriptionally active regions outside of known gene annotations when added to the Chromium workflow (Supplementary Fig. 7d). We found that STRS captured many RNAs that were not present in Visium libraries. Many of these features map outside of or antisense to known annotations (Fig. 1c). We also found that STRS detected many noncoding transcripts that are intragenic to other genes (Fig. 1c). Standard short-read sequencing was sufficient to delineate these features from the surrounding host genes, as reflected by the expression count matrices for STRS versus Visium data (Fig. 1d). Most importantly, we were able to spatially map each of these features and visualize spatial patterns of gene expression (Fig. 1e). We found that features that were incompletely annotated (*ENSMUSG00002075551*) showed sparse spatial expression. Several highly abundant genes showed homogenous patterns of expression, reflecting putative (*Gm42826*) or known (*7SK*) housekeeping roles^10^.

We also asked whether in situ polyadenylation enables capture of non-A-tailed viral RNA. To this end, we assayed murine heart tissues infected with Type 1-Lang reovirus (REOV), a segmented double-stranded RNA virus that expresses ten transcripts that are not polyadenylated. No REOV transcripts were detected with the Visium workflow, whereas STRS enabled detection of more than 200 UMIs representing all ten REOV gene segments (Fig. 1f). To deeply profile viral RNAs, we performed targeted enrichment of viral-derived cDNA from the final sequencing libraries and resequenced the products. This enrichment led to a further increase of around 26-fold of the mean viral UMIs captured per spot (minimum L1 segment with 262 UMIs, maximum S4 segment with 1095 UMIs). Taken together, these findings demonstrate that STRS enables the study of many types of RNAs that are not detectable with existing technologies.

### STRS reveals spatial gene regulation in muscle regeneration

**Fig. 2:**
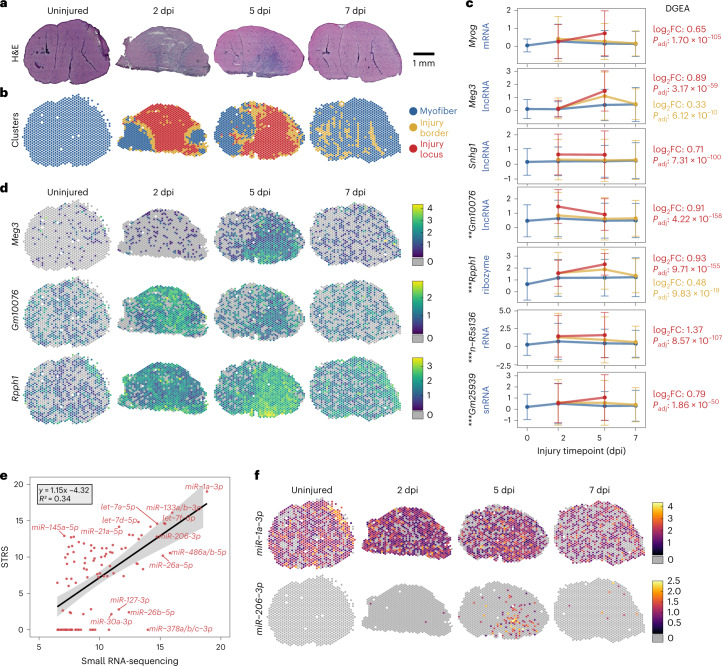
Spatial total RNA-sequencing of regenerating skeletal muscle. **a**, H&E histology of mouse tibialis anterior muscles collected 2, 5 and 7 dpi. **b**, Clustering of spot transcriptomes based on total transcriptome repertoires (Methods). **c**, Differentially expressed RNAs across regional clusters. The *y* axis shows log-normalized expression of each feature. Mean expression across each cluster is reported, colored according to the legend in **b**. Error bars show s.d. Reported statistics to the right of plots reflect differential gene expression analysis performed across clusters on merged STRS samples (*n* = 4,257 spots from four tissue sections; two-sided Wilcoxon Rank-Sum test; Methods). Asterisks next to transcript names reflect differential expression analysis performed across skeletal muscle Visium (*n* = 2,806 spots from three tissue sections) and STRS samples showing adjusted *P* value (*P*_adj_) (**P_adj_ < 10-50, ***P_adj_ < 10–150; two-sided Wilcoxon Rank-Sum test; Methods). FC, fold change. **d**, Spatial maps for select features from **c**. Color sale indicates log-normalized expression, or that the transcript was not detected (gray). **e**, Average detection of miRNAs compared between small RNA-sequencing (*n* = 8) and STRS (*n* = 4). Axes show log_2_ counts per million transcripts, normalized to the total number of transcripts that map to small RNA loci with miRge3.0 (Methods). The top 100 most abundant miRNAs detected by small RNA-sequencing are shown. Line shows a linear regression and 95% confidence interval. **f**, Spatial maps of mature miRNA expression detected by STRS. Color scale shows log-normalized miRNA counts, quantified by miRge3.0. Gray indicates spots in which the transcript was not detected.

Skeletal muscle regeneration is a coordinated system guided by complex gene regulatory networks^5,11–15^. We applied STRS to spatially map the coding and noncoding transcriptome in a mouse model of skeletal muscle regeneration. We injured tibialis anterior muscles and then collected tissues at 2, 5 and 7 days postinjury (dpi) in addition to an uninjured control (Methods). Hematoxylin and eosin (H&E) imaging showed immune infiltration centrally within tissue sections at 2 and 5 dpi, which was mostly resolved by 7 dpi (Fig. 2a). Unsupervised clustering identified spots in the injury loci, spots around the border of the injury loci and spots under intact myofibers (Fig. 2b; Methods).

We performed differential gene expression analysis across the regional clusters to identify noncoding RNAs specific to the injury locus (Fig. 2c; Methods). We found several RNAs that were spatiotemporally associated with injury locus, many of which are undetected or poorly detected by Visium (Fig. 2c,d). *Meg3* is an endogenously polyadenylated lncRNA that has been shown to regulate myoblast differentiation in vitro. We found *Meg3* expression to be confined to the injury locus at 5 dpi, when myoblast differentiation and myocyte fusion occurs^5,16^. *Gm10076*, a transcript with a biotype annotation conflict (Ensembl: lncRNA; NCBI: pseudogene) and no known function, was highly and specifically expressed within the injury locus at 2 dpi. *Gm10076* expression was reduced but still localized to the injury site by 5 dpi and returned to baseline levels by 7 dpi. *Rpph1*, a ribozyme and component of the RNase P ribonucleoprotein which has also been shown to play roles in tRNA and lncRNA biogenesis^17,18^, showed broad expression by 2 dpi that peaked and localized to the injury site at 5 dpi. We also found that STRS captured high levels of antisense transcripts for *Rpph1*, which were not detected by the Visium chemistry. This demonstrates that STRS can robustly profile both polyadenylated and nonpolyadenylated RNAs across heterogeneous tissues.

We next performed cell-type deconvolution using BayesPrism^5,19^ on each spot individually for the Visium and STRS skeletal muscle datasets (Methods). We found that the computed cell-type spatial distributions across each STRS sample were similar to the corresponding Visium sample from the same injury timepoint (Supplementary Fig. 8a). To further assess the similarity of the spot deconvolution achieved for the STRS and Visium data, we merged the STRS and Visium spots and performed principal component analysis on cell-type fractions for all 25 cell types in the single-cell reference (theta values, BayesPrism). We found that the cell-type profiles associated based on the spot gene expression cluster and the injury timepoint and showed similar patterns regardless of the method used (Supplementary Fig. 8b–d). We finally compared mean cell-type fractions across paired samples and found high concordance between Visium and STRS (Supplementary Fig. 8e).

The role of miRNAs in skeletal muscle regeneration has been well established^14,20–22^. Mature miRNAs^23^ are around 22 nucleotides long, are not polyadenylated and are not captured by the standard Visium workflow (Supplementary Fig. 9). We asked whether STRS was able to detect mature miRNAs. We generated matched bulk small RNA-sequencing libraries from entire tibialis anterior muscles as a gold-standard reference (*n* = 2 per timepoint). We used miRge3.0 (ref. ^24^) to quantify mature miRNA abundance in the STRS and matched small RNA-sequencing libraries (Methods). We found similarities in the abundance of the most highly expressed miRNAs between STRS and small RNA-sequencing, but saw drop-out of lowly expressed miRNAs (Fig. 2e and Supplementary Fig. 9). This drop-out is probably due to length biases in the cDNA and DNA sequencing library preparation reactions. We identified many examples of mature miRNA expression in STRS data, including expression of classic ‘myomiRs’, *miR-1a-3p*, *miR-133a/b-3p* and *miR-206-3p* (Fig. 2f)^25^. Consistent with previous studies^26^, we detected static expression of *miR-1a-3p* across all four timepoints (Fig. 2d), whereas *miR-206-3p* was highly expressed within the injury locus at 5 dpi, with very low levels of expression detected at other timepoints.

### STRS spatially resolves viral infection of the murine heart

**Fig. 3:**
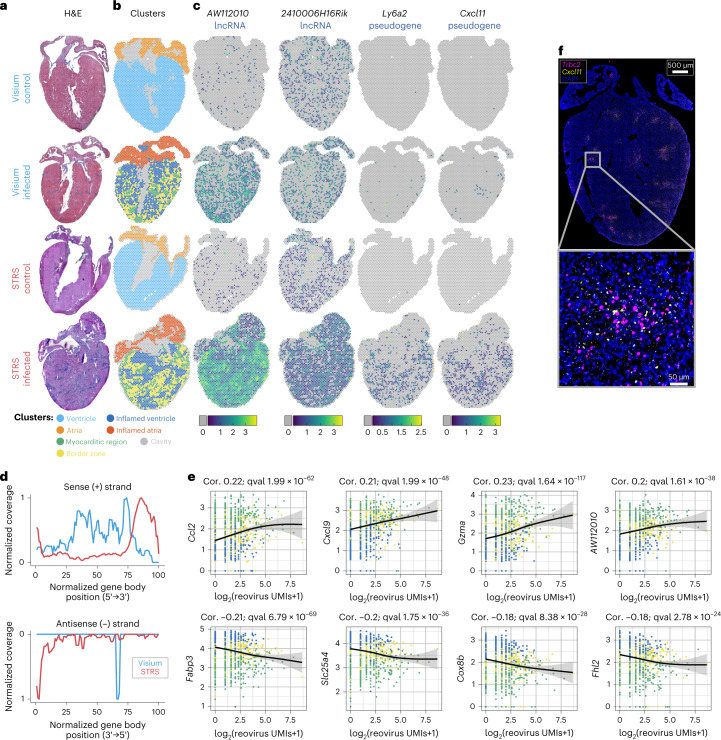
STRS enables simultaneous analysis of viral infection and host response. **a**, H&E staining of control and REOV-infected hearts, collected using the standard Visium workflow and STRS. **b**, Tissue regions identified through unsupervised clustering of spot transcriptomes. **c**, Log-normalized expression of noncoding and coding RNAs that are highly expressed in myocarditic regions. Spots in which transcripts were not detected are shown in gray. **d**, Normalized coverage of deduplicated reads for the sense (+) and antisense (–) strands of all ten REOV gene segments. The *x* axis shows the length-normalized position across the gene bodies of all ten REOV segments. Note that the peak in antisense (–) coverage for the Visium sample (blue) corresponds to only 11 total reads. **e**, Co-expression of pulldown-enriched REOV UMIs versus infection-associated genes in spots underneath inflamed and myocarditic tissue. Spots are colored according to legend in **b**. The line shows log_2_-normalized REOV counts (*x* axis) and log_10_-normalized gene expression (*y* axis) fit to a general additive model and error bands show a 95% confidence interval (Methods). Correlation (Cor.) and *Q* value (qval) reported are from general additive model analysis. **f**, Multiplexed RNA-smFISH (*n* = 2 replicates; Methods) for the T cell marker *Trbc2* and the pseudogene *Cxcl11* in an infected heart.

We next explored the potential for STRS to profile host–virus interactions in a mouse model of viral-induced myocarditis. We orally infected neonatal mice with type 1-Lang REOV, a double-stranded RNA virus with gene transcripts that are not polyadenylated. Within 7 days of oral infection, REOV spreads to the heart and causes myocarditis^27–29^. We performed Visium and STRS on hearts collected from REOV-infected and saline-injected control mice (Fig. 3a,b). We found that REOV transcripts were detected only in the infected heart via STRS and that targeted enrichment of REOV transcripts enabled deeper profiling of viral infection (Figs. 1d and 3a; Methods). Mapping these reads across the tissue revealed pervasive infection across the heart (1,329/2,501 or 53% spots under the tissue; Fig. 1d). Foci containing high viral UMI counts overlapped with myocarditic regions as identified by histology.

We next compared the read coverage profiles across the ten REOV gene segments for REOV-enriched libraries from Visium and STRS samples (Fig. 3d). As expected, STRS libraries had a peak in coverage at the 3′ end of viral gene segments. In contrast, the REOV-enriched Visium reads contained peaks in the middle of viral gene segments as expected for a chemistry that relies on the spurious capture of viral RNA at poly(A) repeats within the transcripts^30^. Interestingly, we found that STRS led to an overrepresentation of reads from the 5′ end of the sense (+) strand of all ten REOV segments. These reads may represent incomplete transcripts generated by transcriptional pausing of the REOV RNA polymerase or transcripts undergoing 3′ exonucleolytic degradation. Finally, we detected the 3′ end of the antisense (−) strand for nine of the ten segments of the REOV genome, suggesting that STRS captures both strands of the dsRNA REOV genome (Fig. 3d). These antisense reads were present at an average ratio of around 1:40 compared with the sense reads. The current model for synthesis of REOV dsRNA posits that dsRNA synthesis only occurs within a viral core particle after packaging of the ten viral positive-sense RNAs. There are several possible explanations for our detection of the antisense strands. One is that we are detecting negative-strand viral RNA that is part of dsRNA that has been released from damaged viral particles either within the cytoplasm or within lysosomes. dsRNA released within endolysosomes can be transported into the cytoplasm by RNA transmembrane receptors SIDT1 and SIDT2 (refs. ^31,32^). A second possibility is that antisense (−) viral RNA is synthesized before packaging of dsRNA into viral particles.

Because STRS efficiently recovers viral RNA, we were able to directly correlate host transcriptomic responses with viral transcript counts for spots in inflamed regions (generalized additive model; Methods). We found inflammation-associated cytokine transcripts such as *Ccl2* and *Cxcl9*, and immune cell markers such as *Gzma* and *Trbc2* to be upregulated in spots with high viral counts (Fig. 3e). We continued this analysis by performing unsupervised clustering (Fig. 3b) and differential gene expression analysis to identify transcripts associated with infection that are more readily detected by STRS (Fig. 3c). *AW112010*, which has been shown recently to regulate inflammatory T cell states^33^, was found only in infected samples and was more abundant in the STRS data compared with Visium. We also found that STRS led to increased detection of putative protein-coding genes, including *Ly6a2*, *Cxcl11* and *Mx2*, which were associated with infection. Interestingly, all three genes are annotated as pseudogenes in GENCODE annotations but have biotype conflicts with other databases. The increased abundance as measured by STRS could reflect differential mRNA polyadenylation for these transcripts. We further validated the localization of *Cxcl11* to REOV infection loci using multiplexed single-molecule RNA fluorescence in situ hybridization (Methods). We found that *Cxcl11* localizes around the T cell marker *Trbc2* in REOV-infected heart tissue, which is specific to infection loci^4^ (Fig. 3f and Supplementary Fig. 10). We also performed spot deconvolution with BayesPrism using a matched single-cell dataset as a reference^4^. We then directly correlated viral transcript counts with estimated cell-type fraction and found that infection-associated cell types (including T cells, dendritic cells, endothelial cells and natural killer cells) correlate with REOV transcript abundance (Supplementary Fig. 11). Overall, STRS enabled more robust, spatially mapped analysis of the host response to infection by increasing the breadth of captured transcript types and by providing direct comparison with viral transcript abundance.

## Discussion

Here, we demonstrate in situ polyadenylation of RNA in sectioned tissues to enable STRS. Enzymatic polyadenylation is frequently implemented for bulk sequencing of total RNA and was recently adopted for single-cell RNA-sequencing^6,7^. STRS implements in situ RNA-labeling for spatial total RNA-sequencing.

STRS has several notable features. First, STRS is compatible with a commercial workflow and requires the use of only one additional reagent. STRS can be adopted easily by others as it requires minimal additional experimental time (around 30 min) and cost and does not require any specialized equipment. We find that the manufacturer-recommended Visium sequencing depths enable effective analysis of captured RNAs in STRS (Supplementary Figs. 1f and 3); however, we recommend that STRS samples be allocated around 10–20% more sequencing reads per spot covered by tissue to account for the expanded repertoire of captured RNAs. Second, because our RNA-labeling strategy is designed to work with poly(dT) reverse transcription, STRS is probably compatible with other sequencing-based spatial transcriptomics platforms. The resolution of our analyses is limited by the size and distribution of the barcoded spots on the Visium slides. Future iterations of STRS that use higher resolution RNA-capture platforms, including Slide-SeqV2 (ref. ^34^), BGI Stereo-seq^35^ or new versions of Visium, promise substantial improvements in spatial resolution. Because STRS is not targeted and does not require previous sequence information, it is easily adapted to new biological systems and is well suited for assaying unknown RNAs, including new RNAs or nonhost transcripts. We investigated the utility and versatility of STRS by applying it to two distinct models. First, we profiled the noncoding RNA repertoires of infiltrating immune cells and regenerating myogenic cells at injury loci in mouse muscle. Second, we analyzed the host transcriptome in response to mammalian orthoreovirus infection. Members of the Reoviridae family of viruses synthesize nonpolyadenylated viral mRNAs, as do arenaviruses and flaviviruses^36–38^. Because STRS can directly capture viral RNAs, we could directly compare viral RNA abundance with gene expression changes in heart tissue. This enabled identification of infection-related noncoding RNAs that were not detectable using standard techniques. Adding spatial context has clarified the underlying biology of gene expression measurements. STRS improves on these facets by extending the assayable transcriptome and enabling direct measurements of viral-derived RNA transcripts.

With STRS, we demonstrated a method to simultaneously map miRNAs and the mRNAs on which they act. Because of their short length and known biases in adapter ligation, miRNAs are notoriously difficult to assay^39,40^. Furthermore, the Visium Gene Expression protocol uses a tagmentation-based library preparation that depletes short molecules by either cutting the UMI/spot barcode or by producing a read that is too short to confidently align to the genome. Despite these issues, we showed robust detection for several known myomiRs and strong correlation with a gold-standard bulk method that does not suffer from ligation or length biases. With future improvements to the library preparation strategy, many of these hurdles can be further reduced.

This work highlights opportunities for improvements in current bioinformatic tools and resources for single-cell and spatial transcriptomics. Current alignment and transcript counting tools are not optimized for total RNA data and genome annotations are incomplete outside of protein coding genes. Furthermore, new tools that go beyond UMI counts and better leverage the wealth of information in sequence read alignment patterns are likely to be impactful.

## Methods

### Mice

The Cornell University Institutional Animal Care and Use Committee (IACUC) approved all animal protocols and experiments were performed in compliance with its institutional guidelines. For skeletal muscle samples, adult female C57BL/6J mice were obtained from Jackson Laboratories (catalog no. 000664) and were used at 6 months of age. For heart samples, confirmed pregnant female C57BL/6J mice were ordered from Jackson Laboratories to be delivered at embryonic stage E14.5.

### Viral infection

Litters weighing 3 g per pup were orally gavaged using intramedic tubing (Becton Dickinson, calalog no. 427401) with 50 μl with 10^7^ plaque-forming units of REOV type 1-lang (T1L) strain in 1× phosphate buffered saline (PBS) containing green food color (McCormick) via a 1 ml tuberculin slip tip syringe (BD, catalog no. 309659) and 30G × 1/2 needle (BD, catalog no. 305106). Litters treated with 1× PBS containing green food color alone on the same day were used as mock controls for the respective infection groups. The mock-infected and REOV-infected pups were monitored and weighed daily until the timepoints used in the study (7 dpi). After dissection, samples were embedded in OCT Compound (Tissue-Tek) and frozen fresh in liquid nitrogen.

### Muscle injury

To induce muscle injury, both tibialis anterior muscles of 6-month-old C57BL/6J mice were injected with 10 µl notexin (10 µg ml^–1^; Latoxan). Either before injury or 2, 5 or 7 dpi, mice were sacrificed and tibialis anterior muscles were collected. After dissection, samples were embedded in OCT Compound (Tissue-Tek) and frozen fresh in liquid nitrogen.

### In situ polyadenylation and STRS

STRS was performed using a modified version of the Visium protocol. Tissue sections (10 μm thick) were mounted onto the Visium Spatial Gene Expression v1 slides. For heart samples, one tissue section was placed into each 6 × 6mm capture area. For skeletal muscle samples, two tibialis anterior sections were placed into each capture area. After sectioning, tissue sections were fixed in methanol for 20 min at −20 °C. Next, H&E staining was performed according to the Visium protocol, and tissue sections were imaged on a Zeiss Axio Observer Z1 Microscope using a Zeiss Axiocam 305 color camera. H&E images were shading corrected, stitched, rotated, thresholded and exported as TIFF files using Zen v.3.1 software (Blue edition). After imaging, the slide was placed into the Visium Slide Cassette. In situ polyadenylation was then performed using yeast poly(A) polymerase (yPAP; Thermo Scientific, catalog no. 74225Z25KU). First, samples were equilibrated by adding 100 µl 1× wash buffer (20 µl 5× yPAP Reaction Buffer, 2 µl 40 U µl^–1^ Protector RNase Inhibitor, 78 µl nuclease-free H_2_O) (Protector RNase Inhibitor; Roche, catalog no. 3335402001) to each capture area and incubating at room temperature for 30 s. The buffer was then removed. Next, 75 µl yPAP enzyme mix (15 µl 5× yPAP reaction buffer, 3 µl 600U µl^–1^ yPAP enzyme, 1.5 µl 25 mM ATP, 3 µl 40U µl^–1^ Protector RNase Inhibitor, 52.5 µl nuclease-free H_2_O) was added to each reaction chamber. STRS was also tested with 20 U µl^–1^ SUPERase-In RNase Inhibitor (Thermo Fisher Scientific, catalog no. AM2694), but we found that SUPERase was not able to prevent degradation of longer transcripts during in situ polyadenylation (Supplementary Fig. 12). The reaction chambers were then sealed, and the slide cassette was incubated at 37 °C for 25 min. The enzyme mix was then removed. Before running STRS, optimal tissue permeabilization time for both heart and skeletal muscle samples was determined to be 15 min using the Visium Tissue Optimization Kit from 10x Genomics. Following in situ polyadenylation, the standard Visium library preparation was followed to generate cDNA and final sequencing libraries. The libraries were then pooled and sequenced according to guidelines in the Visium Spatial Gene Expression protocol using either a NextSeq 500 or NextSeq 2000 (Illumina).

### Targeted pulldown enrichment of viral fragments

We performed hybridization-based enrichment of viral fragments on the Visium and STRS libraries for REOV-infected hearts using the xGen Hybridization and Wash Kit (IDT; 1080577)^4^. In this approach, a panel of 5′-biotinylated oligonucleotides was used for capture and pulldown of target molecules of interest, which were then PCR amplified and sequenced. We designed a panel of 202 biotinylated probes tiled across the entire REOV T1L genome to selectively sequence viral molecules from the sequencing libraries (Supplementary Table 1). After fragmentation and indexing of cDNA, 300 ng of the final Visium or STRS sequencing libraries from REOV-infected hearts were used for xGen hybridization capture using the xGen NGS Target Enrichment Kit protocol provided by the manufacturer. One round of hybridization capture was performed for the STRS library followed by 14 cycles of PCR amplification. Because of the reduced number of captured molecules, two rounds of hybridization were performed on the Visium libraries. Enriched Visium libraries were PCR amplified for 18 cycles after the first round of hybridization and by 5 cycles after the second round of hybridization. Postenrichment products were pooled and sequenced on the Illumina NextSeq 500.

### Single-nucleus total RNA-sequencing

C2C12 cells were grown to 90% confluence and collected with 0.25% TrypLE (Thermo Fisher Scientific). Nuclei were isolated similar to Petrany et al.^41^. Cells were pelleted by centrifugation at 500*g*, at 4 °C, for 5 min, and resuspended in 6 ml chilled homogenization buffer (0.25 M sucrose, 1% bovine serum albumin, 1× PBS, 0.2 U µl^–1^ SUPERase•In RNase Inhibitor, nuclease-free H_2_O). Then, 1 ml chilled 2.5% Triton-X100 diluted in 1x PBS was added. Cells were incubated on ice for 5 min, then pelleted by centrifugation at 1,000*g* at 4 °C for 5 min. Nuclei were then resuspended in 1× PBS and counted using Trypan blue. A total of 5 million nuclei were suspended in 200 µl 1× PBS, then 800 µl ice-cold methanol was added dropwise to fix. Nuclei were then stored at −20 °C overnight.

On the day of the experiment, nuclei were removed from −20 °C and incubated on ice for 5 min. Nuclei were then pelleted by centrifugation at 1,000*g*, at 4 °C, for 5 min and resuspended in 200 µl wash resuspension buffer (0.04% bovine serum albumin, 1 mM DTT, 0.2 U µl^–1^ SUPERase•In RNase Inhibitor, 3× SSC buffer (Thermo Fisher Scientific, catalog no. 15557044), nuclease-free H_2_O). Nuclei were then pelleted by centrifugation at 1,000*g*, at 4 °C, for 5 min and washed in 200 µl 1× wash buffer (40 µl 5× yPAP reaction buffer, 4 µl 20U µl^–1^ SUPERase•In RNase Inhibitor, 156 µl nuclease-free H_2_O). In situ polyadenylation was then performed by suspending nuclei in 50 µl yPAP enzyme mix (10 µl 5× yPAP Reaction Buffer, 2 µl 600 U µl^–1^ yPAP enzyme, 1 µl 25 mM ATP, 2 µl 20 U µl^–1^ SUPERase•In RNase Inhibitor, 35 µl nuclease-free H_2_O) and incubating at 37 °C for 25 min without agitation. Nuclei were then washed with 500 µl of nuclei suspension buffer^42^ (10 mM Tris-HCl pH 7.5, 10 mM NaCl, 3 mM MgCl_2_, 1% bovine serum albumin, 0.2 U µl^–1^ SUPERase•In RNase Inhibitor, nuclease-free H_2_O) and pelleted by centrifugation at 1,000*g*, at 4 °C, for 5 min. Nuclei were finally resuspended in 200 µl nuclei suspension buffer, counted using a Countess 3 (Thermo Fisher Scientific) and the LIVE/DEAD Viability/Cytotoxicity Kit (Thermo Fisher Scientific, catalog no. L3224), then diluted to the proper concentration. Nuclei for standard single-nucleus RNA-sequencing were processed similarly, but with no in situ polyadenylation step (counted immediately after wash buffer was added). A total of 3,300 nuclei were then loaded onto the Chromium controller (10x Genomics) for a targeted capture of 2,000 nuclei. Libraries were generated using the standard Chromium v.3 protocol. Final libraries were sequenced using the Illumina MiniSeq and Illumina NextSeq 500.

### Small RNA-sequencing

For skeletal muscle samples, following the injury time course, tibialis anterior muscles were dissected and snap frozen with liquid nitrogen. The Norgen Total RNA Purification Kit (catalog no. 17200) was used to extract RNA from 10 mg tissue for each sample. For heart samples, following the infection time course, hearts were dissected, embedded in OCT, and frozen in liquid nitrogen. RNA was extracted with Trizol (Invitrogen, catalog no. 15596026) and glycogen precipitation for a small fraction of each of the heart samples. RNA quality was assessed via High Sensitivity RNA ScreenTape Analysis (Agilent, catalog no. 5067-5579) and all samples had RNA integrity numbers greater than or equal to seven.

Small RNA sequencing was performed at the Genome Sequencing Facility of Greehey Children’s Cancer Research Institute at the University of Texas Health Science Center at San Antonio. Libraries were prepared using the TriLink CleanTag Small RNA Ligation kit (TriLink Biotechnologies). Libraries were sequenced with single-end 50× using a HiSeq2500 (Illumina).

### Preprocessing and alignment of STRS, single-nucleus total RNA-sequencing, Smart-Seq-Total and VASA-seq data

All code used to process and analyze these data can be found at https://github.com/mckellardw/STRS. An outline of the pipelines used for preprocessing and alignment is shown in Supplementary Fig. 1a.

Reads were first trimmed using cutadapt v.3.4 (ref. ^43^) to remove the following sequences: (1) poly(A) sequences from the 3′ ends of reads, (2) the template switch oligonucleotide sequence from the 5′ end of reads derived from the Visium Gene Expression kit (sequence: CCCATGTACTCTGCGTTGATACCACTGCTT), (3) poly(G) artifacts from the 3′ ends of reads, which are produced by the Illumina two-color sequencing chemistry when cDNA molecules are shorter than the final read length and (4) the reverse complement of the template switching oligonucleotide sequence from the 5′ ends of reads (sequence: AAGCAGTGGTATCAACGCAGAGTACATGGG). Next, reads were aligned using either STAR v.2.7.10a^44^ or kallisto v.0.48.0 (ref. ^45^). Workflows were written using Snakemake v.6.1.0 (ref. ^46^).

For STAR, the genomic reference was generated from the GRCm39 reference sequence using GENCODE M28 annotations. For STAR alignment, the following parameters, based on work by Isakova et al. ^6^, were used: outFilterMismatchNoverLmax=0.05, outFilterMatchNmin=16, outFilterScoreMinOverLread=0, outFilterMatchNminOverLread=0, outFilterMultimapNmax=50. Aligned reads were deduplicated for visualization using umi-tools v.1.1.2 (ref. ^47^). Aligned and deduplicated reads were visualized with Integrated Genome Viewer v.2.13.0 (ref. ^48^). Normalized gene position plots and genomic loci profiles were generated using Qualimap v.2.2.2.a^49^.

For kallisto, a transcriptomic reference was also generated using the GRCm39 reference sequence and GENCODE M28 annotations. The default k-mer length of 31 was used to generate the kallisto reference. Reads were pseudoaligned using the ‘kallisto bus’ command with the chemistry set to ‘VISIUM’ and the ‘fr-stranded‘ flag activated to enable strand-aware quantification. Pseudoaligned reads were then quantified using bustools v.0.41.0. First, spot barcodes were corrected with ‘bustools correct‘ using the ‘Visium-v1’ whitelist provided in the Space Ranger software from 10x Genomics. Next, the output bus file was sorted and counted using ‘bustools sort’ and ‘bustools count,’ respectively. To estimate the number of spliced and unspliced transcripts, reads pseudoaligned using kb-python v.0.26.0, using the ‘lemanno’ workflow.

Spots were selected manually based on the H&E images using Loupe Browser from 10x Genomics. Spatial locations for each spot were assigned using the Visium coordinates provided for each spot barcode by 10x Genomics in the Space Ranger software (‘Visium-v1_coordinates.txt’). Downstream analyses with the output count matrices were then performed using Seurat v.4.0.4 (refs. ^50,51^). In addition to manual selection, spots containing fewer than 500 detected features or fewer than 1,000 unique molecules were removed from the analysis. Counts from multimapping features were collapsed into a single feature to simplify quantification. Gene biotype percentages were computed according to gene biotypes provided in the GENCODE M28 annotations.

Single-nucleus data were preprocessed and aligned as described above, with a different barcode whitelist matching the 10x Genomics Chromium v.3 chemistry. Count matrices were filtered for cells with more than 750 unique molecules and less than 5% of reads mapping to mitochondrial genes. Counts were then log-normalized with Seurat. Cells were merged and differential gene expression analysis was performed between the standard and in situ polyadenylated nuclei using the ‘FindMarkers()‘ function. A two-sided Wilcoxon Rank-Sum test was used for differential gene expression analysis.

Raw fastq files for VASA-drop^7^ samples were downloaded from GEO (GSE176588) using parallel-fastq-dump (v.0.6.5). Reads were trimmed using cutadapt v.3.4 to remove poly(A) and poly(G) sequences. Reads were then aligned and quantified using kallisto/BUStools as described above. The ‘–technology’ flag for ‘kallisto bus’ was set to ‘0,6,22:0,0,6:1,0,0’ for cell barcode and UMI identification to reflect the modified fastq files authors uploaded to GEO. Gene counts from multimapping features were collapsed into a single feature.

Raw fastq files for Smart-Seq-Total^6^ samples were downloaded from GEO (GSE151334) using parallel-fastq-dump (v.0.6.5). Reads were then pseudoaligned using ‘kallisto quant’ with the ‘–fragment-length’ flag set to 75 and the ‘–sd’ flag set to ten. Transcript counts were converted to gene counts according to GENCODE M28 gene symbols, then counts from multimapping features were collapsed into a single feature.

### Rarefaction analysis of Visium and STRS data

Raw fastq files for each library were randomly downsampled four times using seqtk v.1.2 to final read counts totaling between 100,000 and 50,000,000 reads. Final libraries were then pseudoaligned using the kallisto pipeline described above.

### Annotation-free quantification of transcriptionally active regions in single-nucleus RNA-sequencing data

The ‘from_STARsolo’ version of the TAR-scRNA-seq^9^ pipeline was used with the outputs from reads aligned with STAR for single-nucleus RNA-sequencing data. Default parameters were used for ‘MERGEBP’ (500) and ‘THRESH’ (10,000,000) for TAR merging and filtering, respectively. Count matrices generated by TAR-scRNA-seq were subset based on cell barcodes that remained after standard quality control.

### Mature microRNA quantification

For STRS data: after trimming (see above), barcode correction with STAR v.2.7.10a and UMI-aware deduplication with umi-tools v.1.1.2, reads were split across all 4,992 spot barcodes and analyzed using miRge3.0 v.0.0.9 (ref. ^24^). Reads were aligned to the miRbase reference provided by the miRge3.0 authors. MiRNA counts were log-normalized according to the total number of counts detected by kallisto and scaled using a scaling factor of 1,000. For small RNAseq data: reads were first trimmed using trim_galore v.0.6.5. Reads were then aligned and counted using miRge3.0 v.0.0.9. Raw fastq files for all skeletal muscle and heart datasets from the Small RNA Atlas^52^ were downloaded from GEO (GSE119661) and processed similarly.

### Unsupervised clustering and differential gene expression analysis of spot transcriptomes

Spot UMI counts as generated by kallisto were used. First, counts were log-normalized and scaled using default parameters with Seurat. Principal component analysis was then performed on the top 2.000 most variable features for each tissue slice individually. Finally, unsupervised clustering was performed using the ‘FindClusters()‘ function from Seurat. The top principal components which accounted for 95% of variance within the data were used for clustering. For skeletal muscle samples, a clustering resolution was set to 0.8. For heart samples, clustering resolution was set to 1.0. Default options were used for all other parameters. Finally, clusters were merged according to similar gene expression patterns and based on histology of the tissue under each subcluster.

Differential gene expression analysis was performed using the ‘FindAllMarkers()‘ function from Seurat. Default parameters were used, including the use of the two-sided Wilcoxon rank-sum test to identify differentially expressed genes. To identify features enriched in the skeletal muscle STRS datasets, all Visium and STRS were first merged and compared according to the method used (Visium versus STRS). To identify cluster-specific gene expression patterns, skeletal muscle samples were first clustered as described above individually. STRS samples were then merged, and differential gene expression analysis was performed across the three injury region groups.

### Cell-type deconvolution of Visium and STRS datasets

Cell-type deconvolution of skeletal muscle Visium and STRS data was performed as previously^5^ using BayesPrism^19^ (previously known as ‘Tumor microEnvironment Deconvolution’, TED, v.1.0; github.com/Danko-Lab/TED). We used the ‘scMuscle’ dataset generated in McKellar et al.^5,53^ as a single-cell transcriptomic reference for skeletal muscle. For heart samples, we used all mock and infected single-cell RNA-sequencing samples generated by Mantri et al.^4^. Highly and differentially expressed genes across cell types were identified with differential gene expression analysis using Seurat (FindAllMarkers, using two-sided Wilcoxon rank-sum test). The resulting genes were filtered based on average log_2_-fold change (avg_logFC > 1) and the percentage of cells within the cluster that express each gene (pct.expressed > 0.5), yielding around 1,000 genes in both single-cell references. Mitochondrial and ribosomal protein genes were removed from this list, in line with recommendations from the BayesPrism authors. For each of the cell types, mean raw counts were calculated across the around 1,000 genes to generate a gene expression profile for BayesPrism. Raw counts for each spot were then passed to the run.Ted function, using the ‘GEP’ option for input.type and default parameters for the remaining inputs. Final Gibbs theta values were used as estimates for the fraction of transcripts from each spot that were derived from each of the cell types. In plots (Supplementary Figs. 8 and 11), a minimum threshold value for theta of 0.01 was used. For skeletal muscle, after deconvolution all spots were merged. Principal component analysis was performed on the nonthresholded BayesPrism theta values using Seurat.

### Correlation analysis between REOV counts, host gene expression and spot cell-type fraction

We used a generative additive model implemented in Monocle v.2.18.0 (ref. ^54^) to find genes that vary with viral UMI count. A Seurat object for STRS data and viral UMI counts from the REOV-infected heart was converted to a CellDataSet object using the ‘as.CellDataSet()’ command implemented in Seurat. For comparison between gene expression and REOV counts, the expression family was set to ‘negative binomial’ as suggested for UMI count data in the Monocle documentation. For comparison between cell-type fraction (theta, as computed by BayesPrism), a minimum theta value of 0.001 was used and the expression family was set to ‘uninormal’. The CellDataSet objects were then preprocessed to estimate size factors and dispersion (just for comparison with genes). Genes expressed in fewer than ten spots were removed. We then used the generative additive model implemented in the ‘differentialGeneTest()’ command in Monocle to identify genes or cell-type fractions that vary with log_2_-transformed viral UMI counts. To find the direction in which these genes varied with viral UMI counts, we calculated the Pearson correlation for all genes with log_2_-transformed viral UMI counts.

### RNA fluorescence in situ hybridization using hybridization chain reaction HCR-V3

Single-molecule fluorescence in situ hybridization (smFISH) was performed as described previously^4,55^. Probes were designed using NCBI primer-blast for two-step hybridization strategy with split probe design and hybridization chain reaction (HCR)-V3 (ref. ^55^) (Supplementary Table 2). Split probes for each gene target were mixed and diluted in nuclease-free water to a final total probe concentration of 10 µM. Hairpin pairs labeled with two different fluorophores, namely Alexa-488 and Alexa-546 (Molecular Instruments), were used for HCR-V3.

Slides with tissue sections were warmed to room temperature and then fixed in 4% paraformaldehyde for 12 min at room temperature. After fixation, sections were washed for 5 mins in 1× PBS twice, incubated for 1 h in 70% ethanol for tissue permeabilization, washed again for 5 mins in 1× PBS, and then used for primary hybridization. Hybridization buffer (HB) mix was prepared with 2× SSC, 5× Denhardt’s solution, 10% ethylene carbonate, 10% dextran sulfate, 0.01% SDS, 1 µM of probe pool mix per target for the hybridization reaction. 20 µl of HB mix (with probes) per section was then put on each slide to cover the tissue section, covered with Parafilm and incubated overnight at 37 °C inside a humidifying chamber for primary hybridization. After primary hybridization, Parafilm was removed and slides were washed in hybridization wash buffer (0.215 M NaCl, 0.02 M Tris-HCl pH 7.5 and 0.005 M EDTA) for 20–30 min at 48 °C. Amplification buffer (AB) mix was prepared with 2× SSC, 5× Denhardt’s solution, 10% dextran sulfate, 0.01% SDS and 0.06 µM HCR hairpins for the amplification reaction. Then, 2 µl of each fluorophore labeled hairpins at 3 µM corresponding to the target genes were mixed, incubated at 95 °C for 1.5 min, covered in aluminum foil and cooled to room temperature for 30 min to form hairpins before adding to the AB mix. A 20 µl portion of AB mix per section was then placed on each slide to cover the tissue section, covered with Parafilm and incubated overnight at room temperature in the dark for signal amplification. After signal amplification, Parafilm was removed and slides were washed in 5× SSCT buffer twice for 30–40 min and then twice for 10 mins. The slides were then cleaned carefully with Kimwipe and treated with Ready Probes Auto-fluorescence Quenching Reagent Mix (Thermo Fisher, catalog no. R37630) for 5 min and washed three times in 1× PBS. Finally, tissue sections were then counterstained with 4,6-diamidino-2-phenylindole for 10 min at room temperature, washed for 5 min in 1× PBS twice, excess PBS was cleaned off using Kimwipe, and sections were immediately mounted on coverslips using Slowfade antifade media, left overnight for treatment and imaged the next day on a Zeiss Axio Observer Z1 Microscope using a Hamamatsu ORCA Fusion Gen III Scientific CMOS camera. FISH images were shading corrected, stitched, rotated, thresholded and exported as TIFF files using Zen v.3.1 software (Blue edition).

### Reporting summary

Further information on research design is available in the Nature Research Reporting Summary linked to this article.

## Online content

Any methods, additional references, Nature Research reporting summaries, source data, extended data, supplementary information, acknowledgements, peer review information; details of author contributions and competing interests; and statements of data and code availability are available at 10.1038/s41587-022-01517-6.

## Supplementary information

Supplementary Information Supplementary Figs. 1–12.

Reporting Summary

Supplementary Table 1 Probes used for targeted sequencing of viral transcripts.

Supplementary Table 2 Probes used in smFISH.

## Data Availability

Previously published spatial RNA-sequencing data were downloaded from Gene Expression Omnibus (GEO) and are available under the following accession numbers; regenerating skeletal muscle^5^ GSE161318 (ref. ^56^), infected heart tissue^4^ GSE189636 (ref. ^57^). Spatial total RNA-sequencing data generated in this study can be found on GEO under the accession number GSE200481 (ref. ^58^). Small RNA-sequencing data are available on GEO under the accession number GSE200480 (ref. ^59^). Single-nucleus RNA-sequencing data for C2C12 nuclei with and without in situ polyadenylation can be found on GEO under the accession number GSE209780 (ref. ^60^). Public datasets for Smart-Seq-Total (GSE151334 (ref. ^61^)), VASA-seq (GSE176588 (ref. ^62^)), the small RNA-sequencing atlas (GSE119661 (ref. ^63^)) and the viral myocarditis single-cell RNA-sequencing reference (GSE189636 (ref. ^57^)) were downloaded from GEO. The skeletal muscle single-cell RNA-sequencing reference was downloaded as a Seurat object from Dryad^5,64^.

## Code availability

A detailed protocol for performing STRS as well as custom analysis scripts for aligning and processing STRS data can be found at https://github.com/mckellardw/STRS^65^.
