# Supplementary material for: Spatial mapping of the total transcriptome by in situ polyadenylation
Source: Nat Biotechnol. 2022 Nov 3;41(4):513–20. doi: 10.1038/s41587-022-01517-6 (PMC10110464; doi:10.1038/s41587-022-01517-6)
Supplement: Supplementary file 1 — Supplementary Figs. 1–12. [file 41587_2022_1517_MOESM1_ESM.pdf]

nature biotechnology

Article https://doi.org/10.1038/s41587-022-01517-6

Spatial mapping of the total transcriptome by in situ polyadenylation

In the format provided by the authors and unedited

Table of Contents

Figure	Page	Title
Figure S1	1	Comparison of bioinformatic analyses for Visium and Spatial Total RNA-Sequencing (STRS)
Figure S2	2	Comparison of transcript capture by biotype between Visium and STRS.
Figure S3	3	Gene-by-gene comparison across Visium and Spatial Total RNA-Sequencing.
Figure S4	4	Transcript biotype spatial distribution comparison between Visium and Spatial Total RNA-Sequencing (STRS) for regenerating mouse skeletal muscle.
Figure S5	5	Transcript biotype spatial distribution comparison between Visium and Spatial Total RNA-Sequencing (STRS) for mouse hearts with and without Reovirus infection.
Figure S6	6	Comparison of gene biotype distributions across single-cell, single-nucleus, and spatial total RNA-sequencing methods.
Figure S7	7	*In situ* polyadenylation enables single-nucleus total RNA-sequencing in C2C12 myoblasts.
Figure S8	8	Spot deconvolution analysis of Visium and STRS data of skeletal muscle during injury response.
Figure S9	9	Comparison of mature microRNA detection in small RNA-sequencing, Visium, and Spatial Total RNA-Sequencing.
Figure S10	10	Single-molecule RNA fluorescence in situ hybridization identifies spatial colocalization of *Cxcl11* to T cell infiltration loci in reovirus-induced myocarditis.
Figure S11	11	Spot deconvolution analysis of Visium and STRS data of mock and reovirus-infected heart samples.
Figure S12	12	DNA fragment analysis of Spatial Total RNA-Sequencing (STRS) cDNA (pre-tagmentation) and final libraries (post-tagmentation).

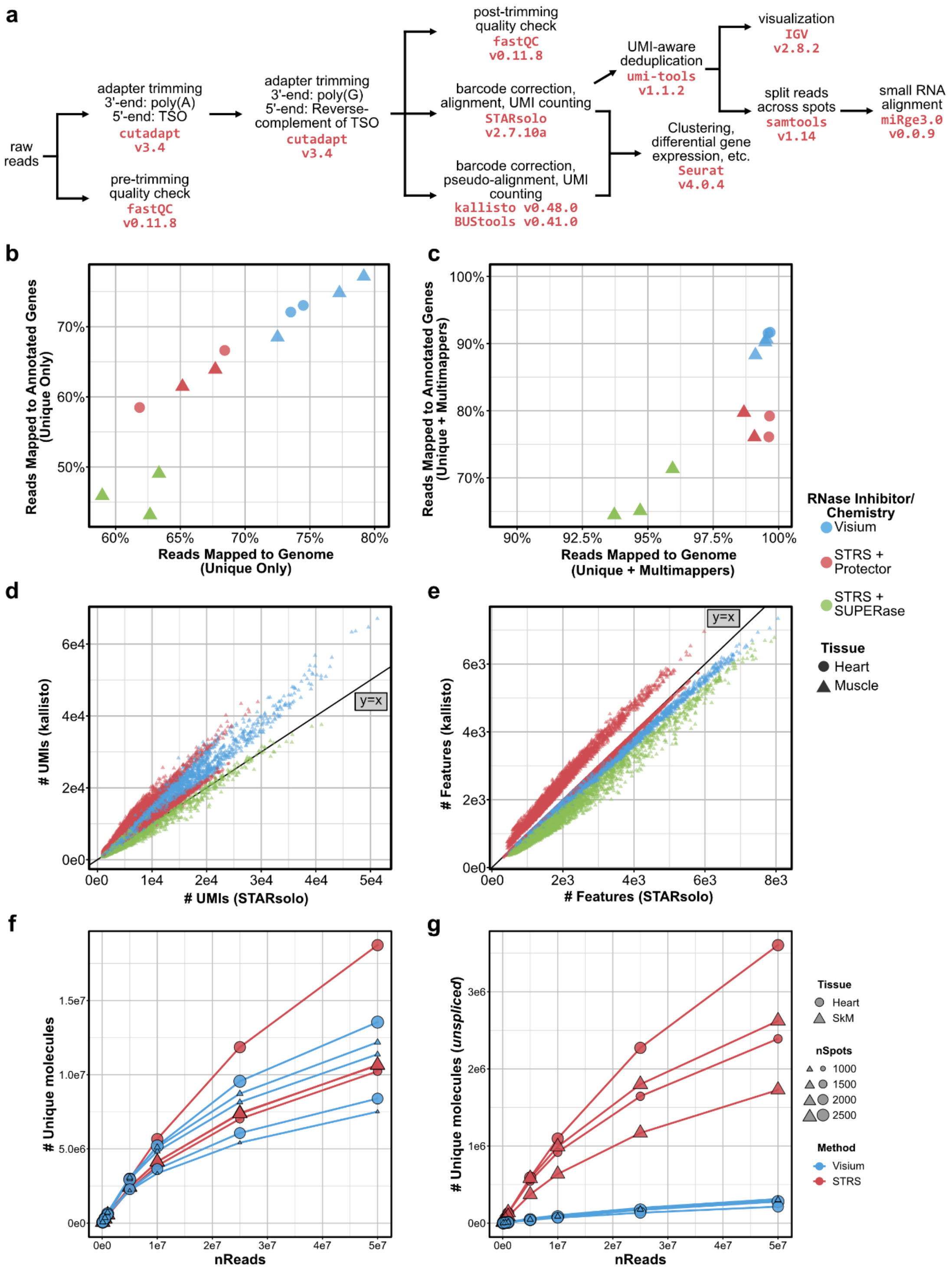

Figure S1. Comparison of bioinformatic analyses for Visium and Spatial Total RNA-Sequencing (STRS). **(a)** Bioinformatic tools and workflows used to preprocess, align, and quantitate transcripts. **(b)** STAR alignment rate for reads mapping to unique genomic position (x-axis) versus reads uniquely mapping to annotated regions (GENCODE M28 annotations) of the genome (y-axis). Each point represents an entire Visium capture area. Points are colored by sample preparation method (see **Methods**) and are shaped according to tissue type. **(c)** STAR alignment rate for reads mapping to unique or multiple positions along the genome (x-axis) versus reads uniquely mapping or multimapping to annotated regions (GENCODE M28 annotations) of the genome (y-axis). Spots are colored as in (b). **(d)** Number of unique molecules (UMIs) detected by STARsolo (x-axis) versus kallisto (y-axis). Each point represents a barcoded spot. Points are colored by sample preparation method (see **Methods**) and are shaped according to tissue type. **(e)** Number of features detected by STARsolo (x-axis) versus kallisto (y-axis). Spots are colored as in (d). **(f)** Rarefaction analysis of Visium and STRS libraries showing the number of unique molecules captured and **(g)** the number of unique unspliced molecules captured at increasing read depths, as quantified by kallisto/BUStools and labelled by tissue and method with marker sizes scaled by number of spots on the capture slide area.

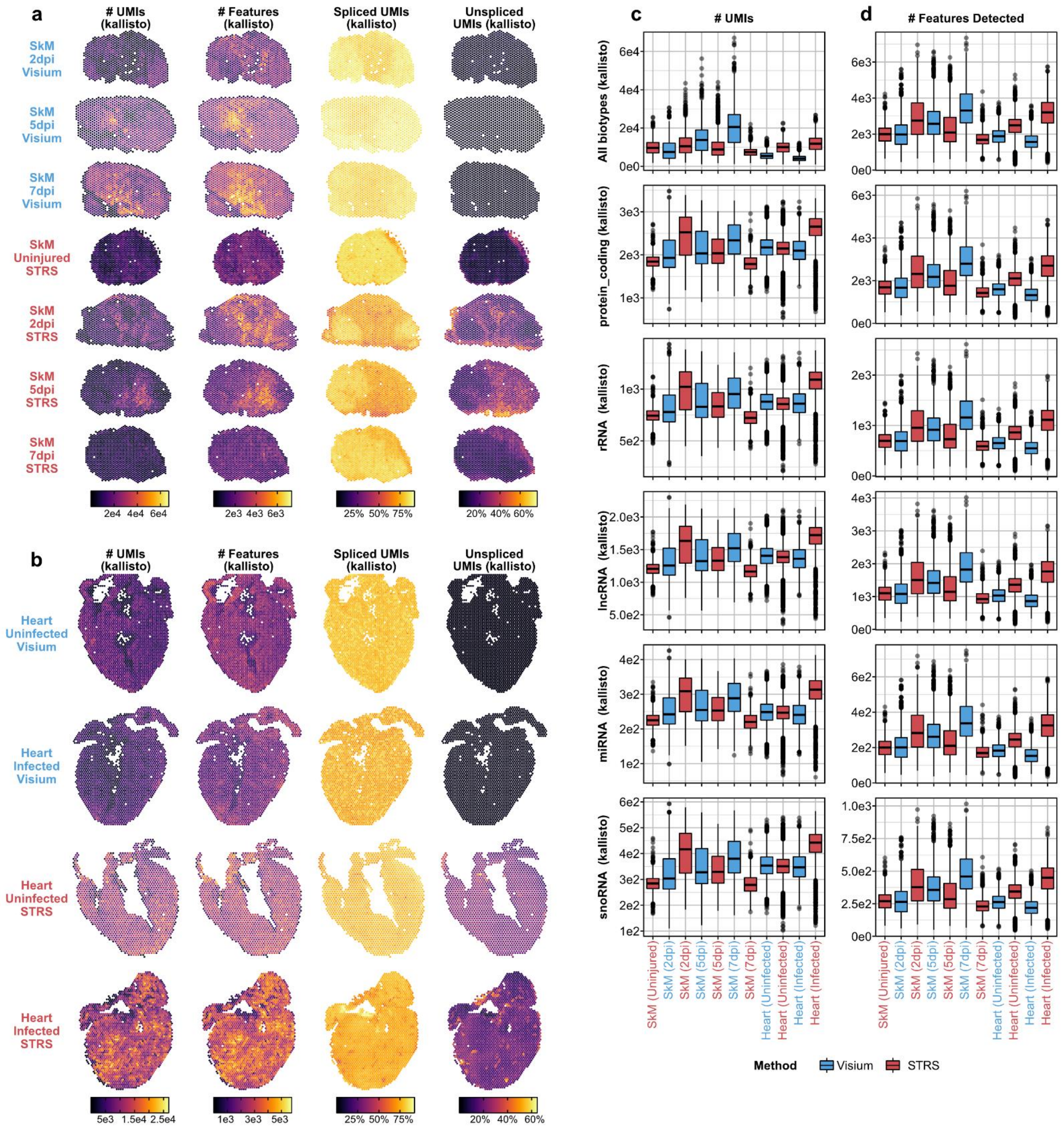

Figure S2. Comparison of transcript capture by biotype between Visium and STRS. **(a)** Spatial maps showing the number of unique molecules (UMIs), the number of features, the number of spliced UMIs, and the number of unspliced UMIs detected for each spot in skeletal muscle samples and **(b)** heart samples. **(c)** Boxplots for the number of UMIs detected and **(d)** the number of features detected in each sample for all genes, protein coding genes, ribosomal RNA (rRNA), long noncoding RNA (lncRNA), micro RNA (miRNA), and small nucleolar RNA (snoRNA). The boxplot shows median and quartile values, and whiskers show 1.5 times interquartile range. Statistics were computed on SkM D2 Visium (n=890 spots), SkM D5 Visium (n=943 spots), SkM D7 Visium (n=973 spots), SkM Uninjured STRS (n=1,045), SkM D2 STRS (n=1,030 spots), SkM D5 STRS (n=1,106 spots), SkM D7 STRS (n=1,076 spots), Heart Uninfected Visium (n=2,593 spots), Heart Infected Visium (n=2,192 spots), Heart Uninfected STRS (n=1,557 spots), and Heart Infected STRS (n=2,333 spots).

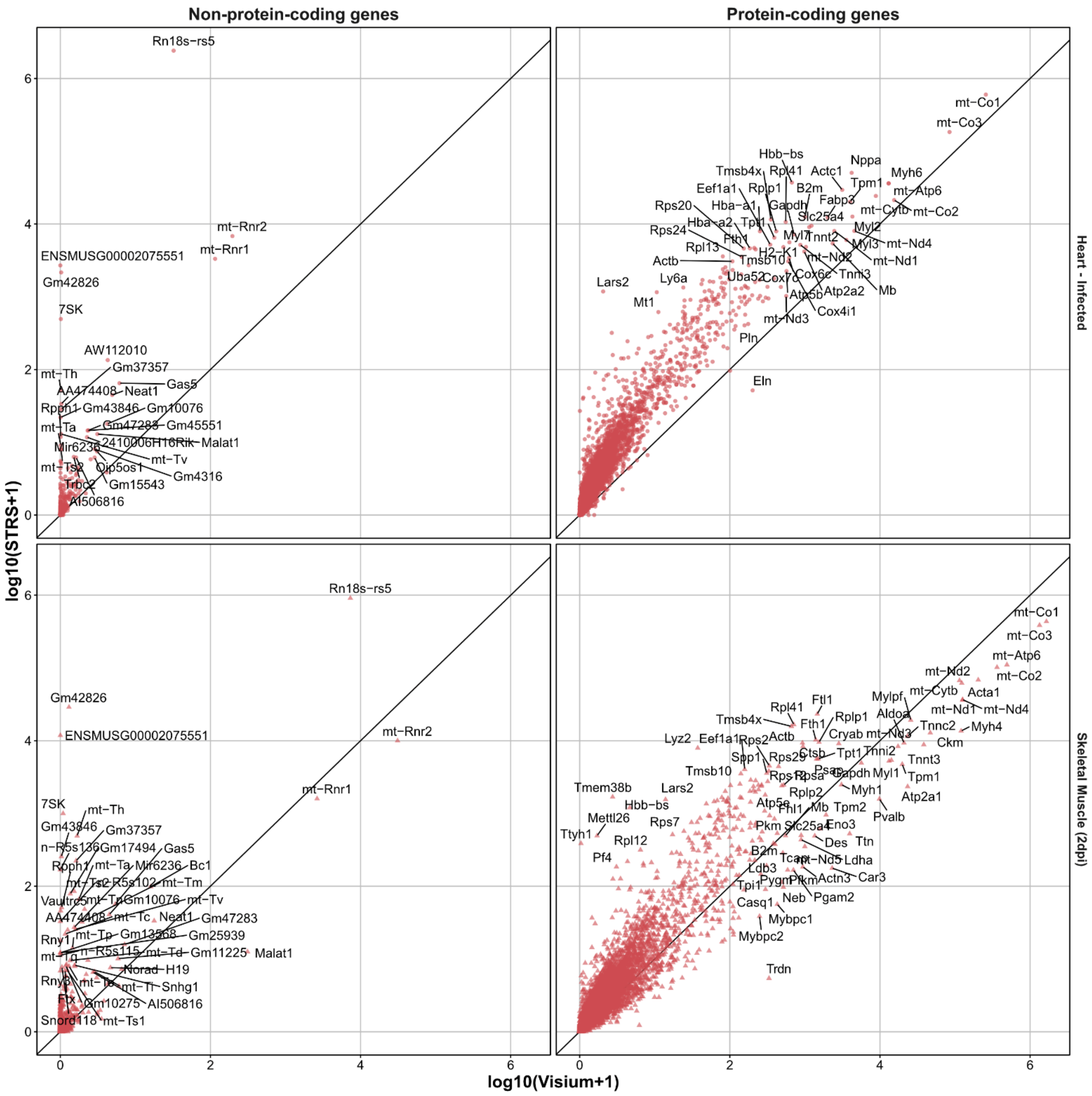

Figure S3. Gene-by-gene comparison across Visium and Spatial Total RNA-Sequencing. Genes are split between protein coding (right) and non-protein-coding (left) genes. Data is shown for injured skeletal muscle (2 days post-injury) and infected heart samples (Fig. 1b-e).

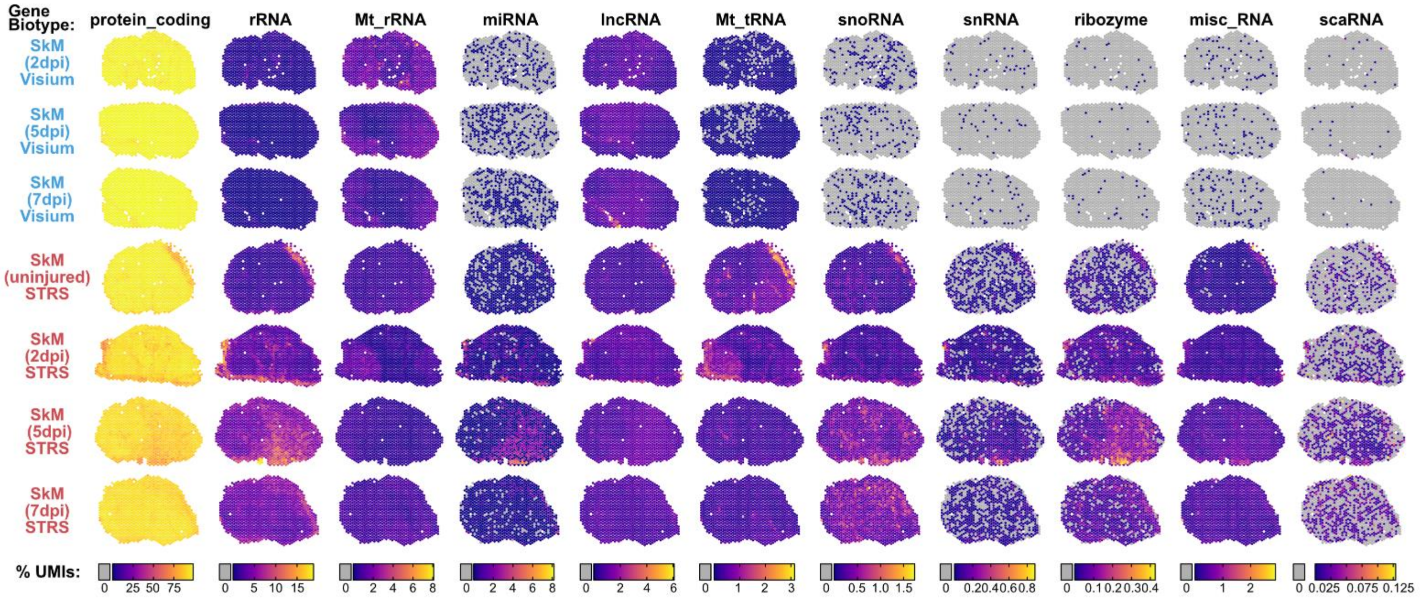

Figure S4. Transcript biotype spatial distribution comparison between Visium and Spatial Total RNA-Sequencing (STRS) for regenerating mouse skeletal muscle. Spatial maps of abundant gene biotypes. Color scale shows the percent of unique molecules (UMIs) for each spot that correspond to each transcript biotype. Gray spots contain no molecules which correspond to the given biotype. Transcript biotypes shown include protein coding, ribosomal RNA (rRNA), mitochondrial ribosomal RNA (Mt_rRNA), microRNA (miRNA), long noncoding RNAs (lncRNA), mitochondrial transfer RNAs (Mt_tRNA), small nucleolar RNA (snoRNA), small nuclear RNA (snRNA), ribozyme, miscellaneous RNA (misc_RNA), and small Cajal body-specific RNA (scaRNA).

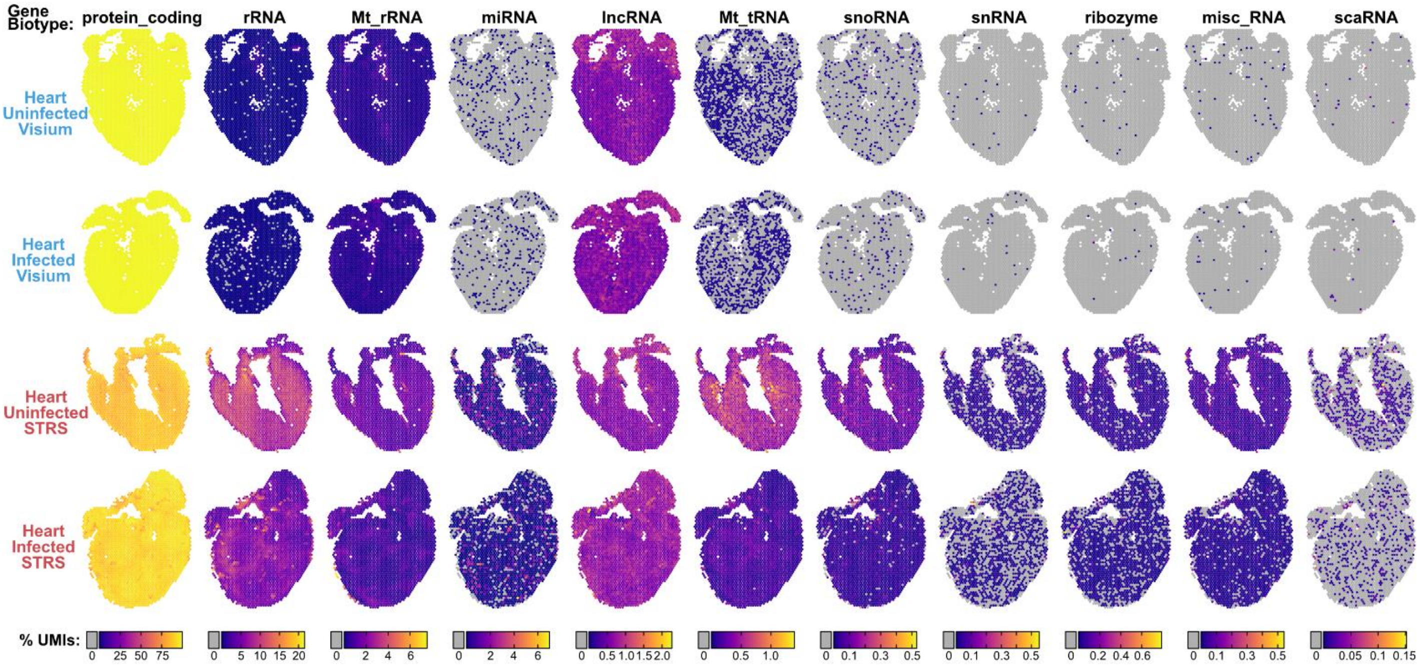

Figure S5. Transcript biotype spatial distribution comparison between Visium and Spatial Total RNA-Sequencing (STRS) for mouse hearts with and without Reovirus infection. Spatial maps of abundant gene biotypes. Color scale shows the percent of unique molecules (UMIs) for each spot that correspond to each transcript biotype. Gray spots contain no molecules which correspond to the given biotype. Transcript biotypes shown include protein coding, ribosomal RNA (rRNA), mitochondrial ribosomal RNA (Mt_rRNA), microRNA (miRNA), long noncoding RNAs (lncRNA), mitochondrial transfer RNAs (Mt_tRNA), small nucleolar RNA (snoRNA), small nuclear RNA (snRNA), ribozyme, miscellaneous RNA (misc_RNA), and small Cajal body-specific RNA (scaRNA).

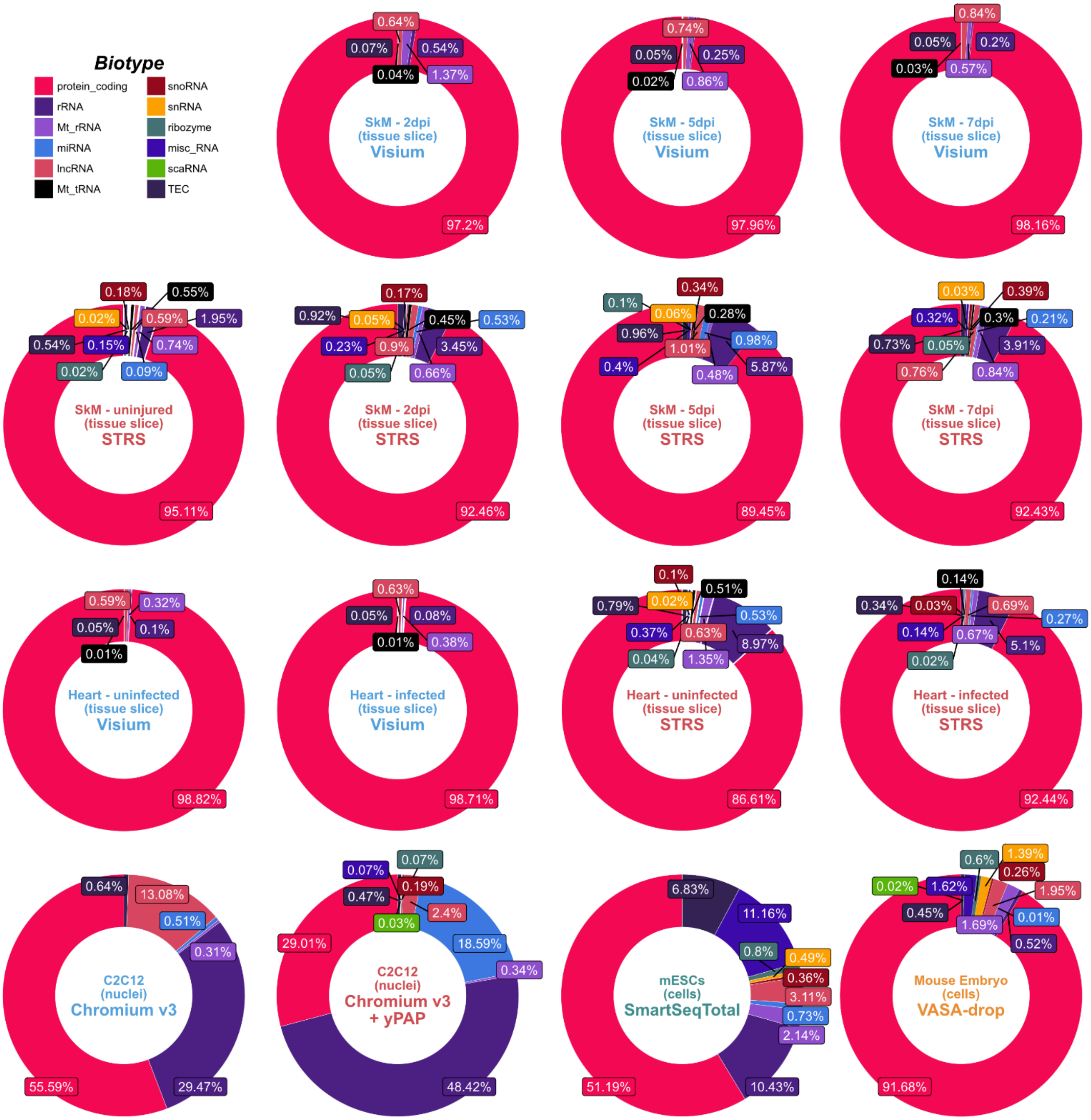

Figure S6. Comparison of gene biotype distributions across single-cell, single-nucleus, and spatial total RNA-sequencing methods. Pie charts of mean unique molecule fractions for each gene biotype which accounts for more than 0.01% of unique molecules across all cells, nuclei, or spots for each sample shown. Transcript biotypes shown include protein coding, ribosomal RNA (rRNA), mitochondrial ribosomal RNA (Mt_rRNA), microRNA (miRNA), long noncoding RNAs (lncRNA), mitochondrial transfer RNAs (Mt_tRNA), small nucleolar RNA (snoRNA), small nuclear RNA (snRNA), ribozyme, miscellaneous RNA (misc_RNA), small Cajal body-specific RNA (scaRNA), and TEC (To be Experimentally Confirmed).

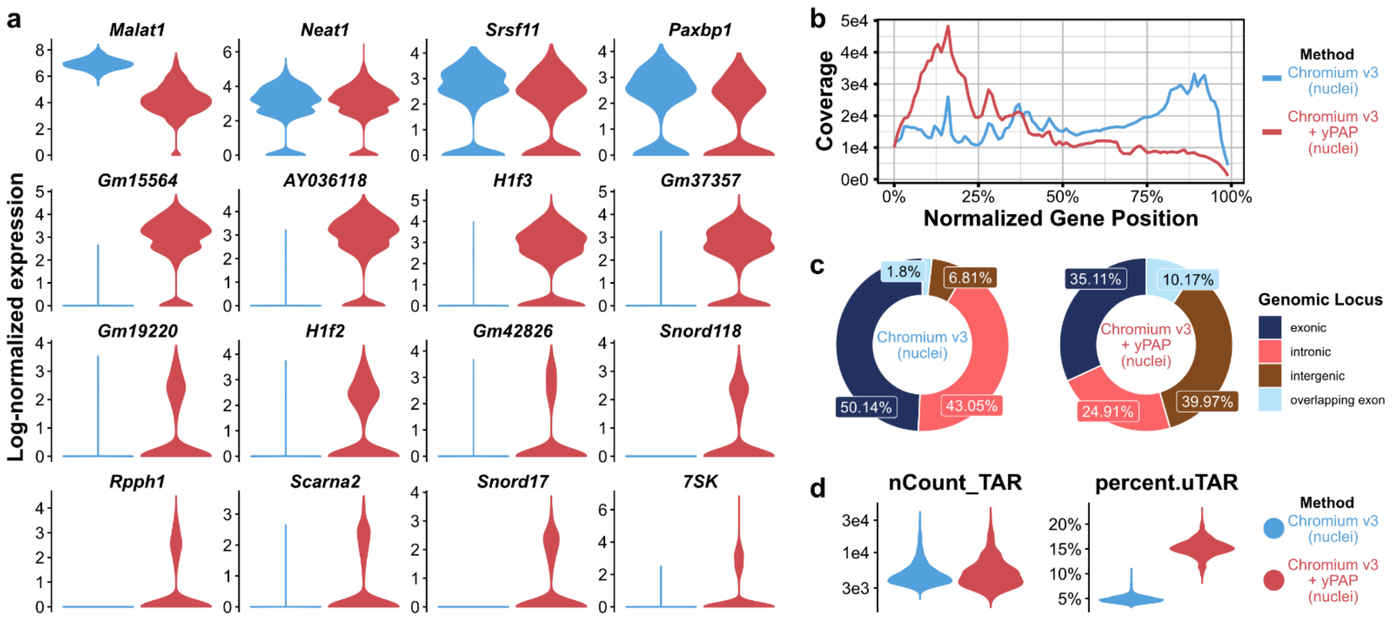

Figure S7. *In situ* polyadenylation enables single-nucleus total RNA-sequencing in C2C12 myoblasts. **(a)** Violin plots showing detection of select features in C2C12 myoblasts using Chromium v3 single-nucleus RNA-sequencing, either with the standard method (blue) or with nuclei preprocessed with yeast poly(A) polymerase (yPAP, red). **(b)** Length-normalized coverage of all genes. **(c)** Genomic locus of aligned reads. **(d)** Violin plots of the number of unique molecules (UMIs) which align to transcriptionally active regions (TARs), as identified by TAR-scRNA-seq (see **Methods**), and the percentage of those UMIs which align to TARs outside of GENCODE M28 annotations (uTARs).

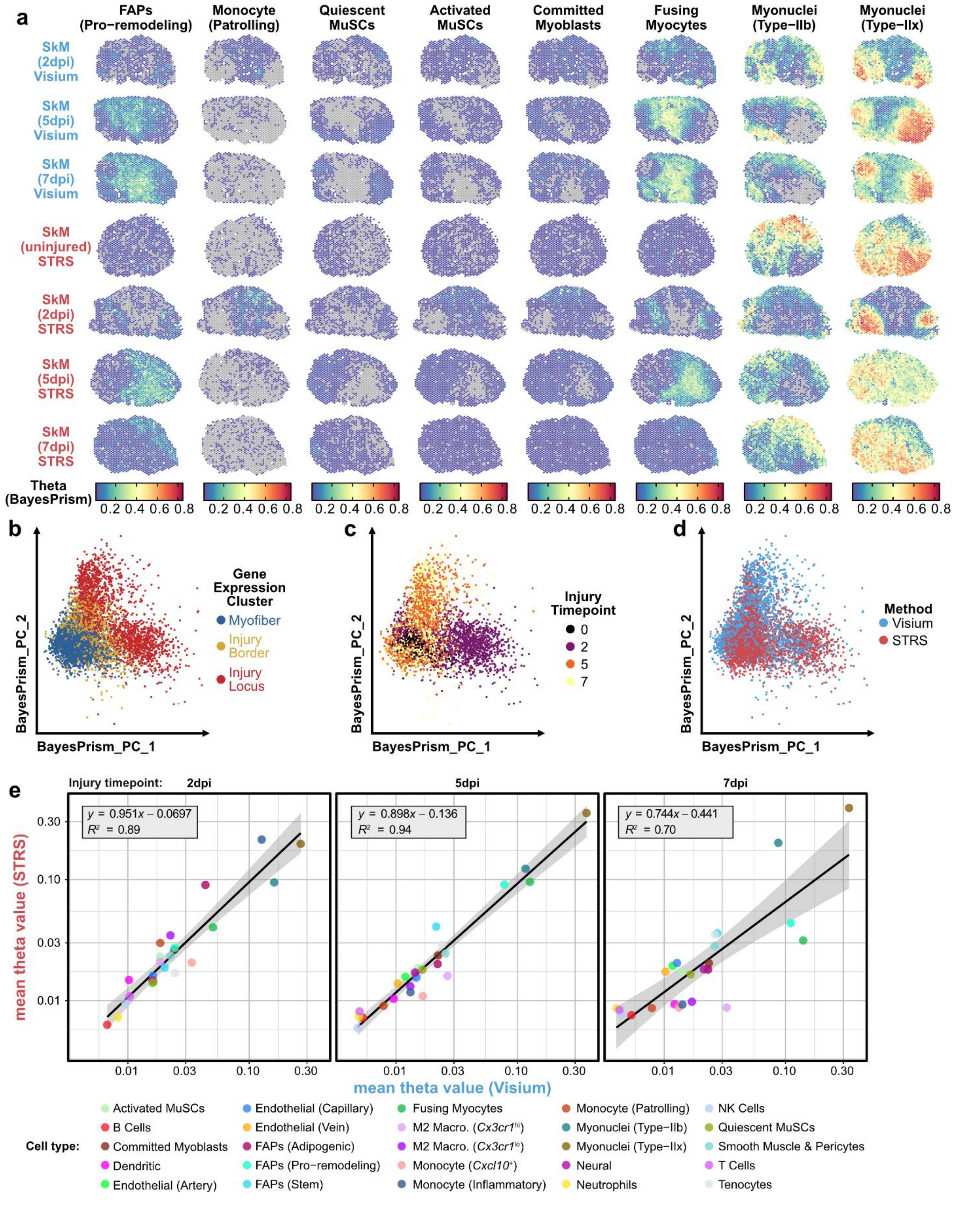

Figure S8. Spot deconvolution analysis of Visium and STRS data of skeletal muscle during injury response. **(a)** Spatial maps of injury-associated cell types. The color scale shows the theta value, or fraction of reads attributed to each cell type, as computed by BayesPrism. **(b)** Scatter plot shows a principal component analysis of the cell type theta values for the merged Visium and STSR data, colored by gene-expression-derived cluster, **(c)** injury timepoint, and **(d)** method used. **(e)** Mean theta values for each cell type, as computed by BayesPrism, for matched injury time points in skeletal muscle samples. Line plot shows a linear regression in black and 95% confidence interval in gray.

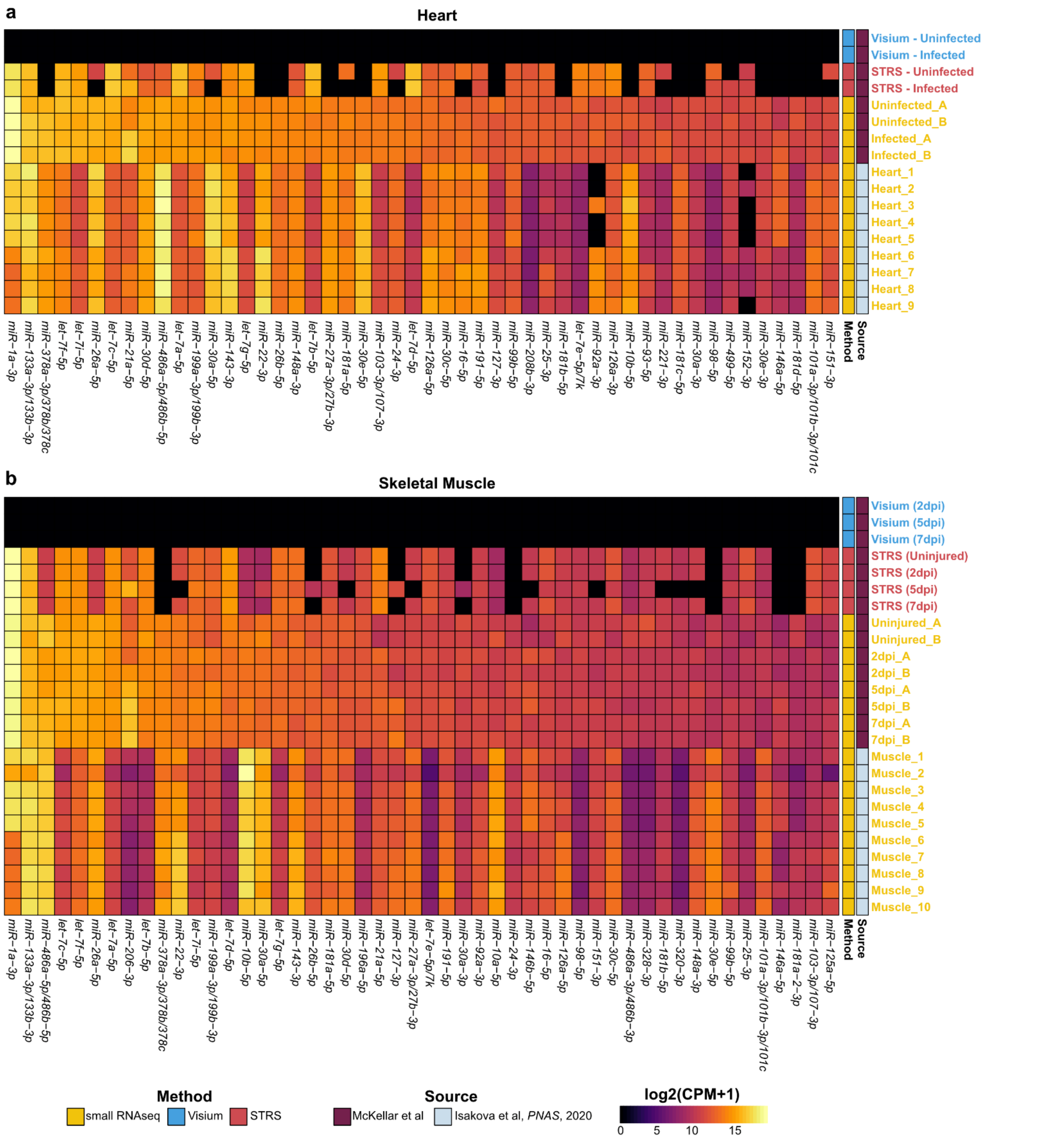

Figure S9. Comparison of mature microRNA detection in small RNA-sequencing, Visium, and Spatial Total RNA-Sequencing. Counts for **(a)** heart samples and **(b)** skeletal muscle samples are shown as log2-transformed counts per million (CPM) with a pseudocount of 1. Counts reflect UMI-deduplicated reads for STRS samples and are normalized to the total number of counts which align to mature microRNAs. Data from sex-matched (female) samples from the mouse tissue atlas of small noncoding RNA are also shown (Isakova et al, *PNAS*, 2020). The miRNAs shown are sorted by average expression within the small RNA-sequencing samples generated in this study.

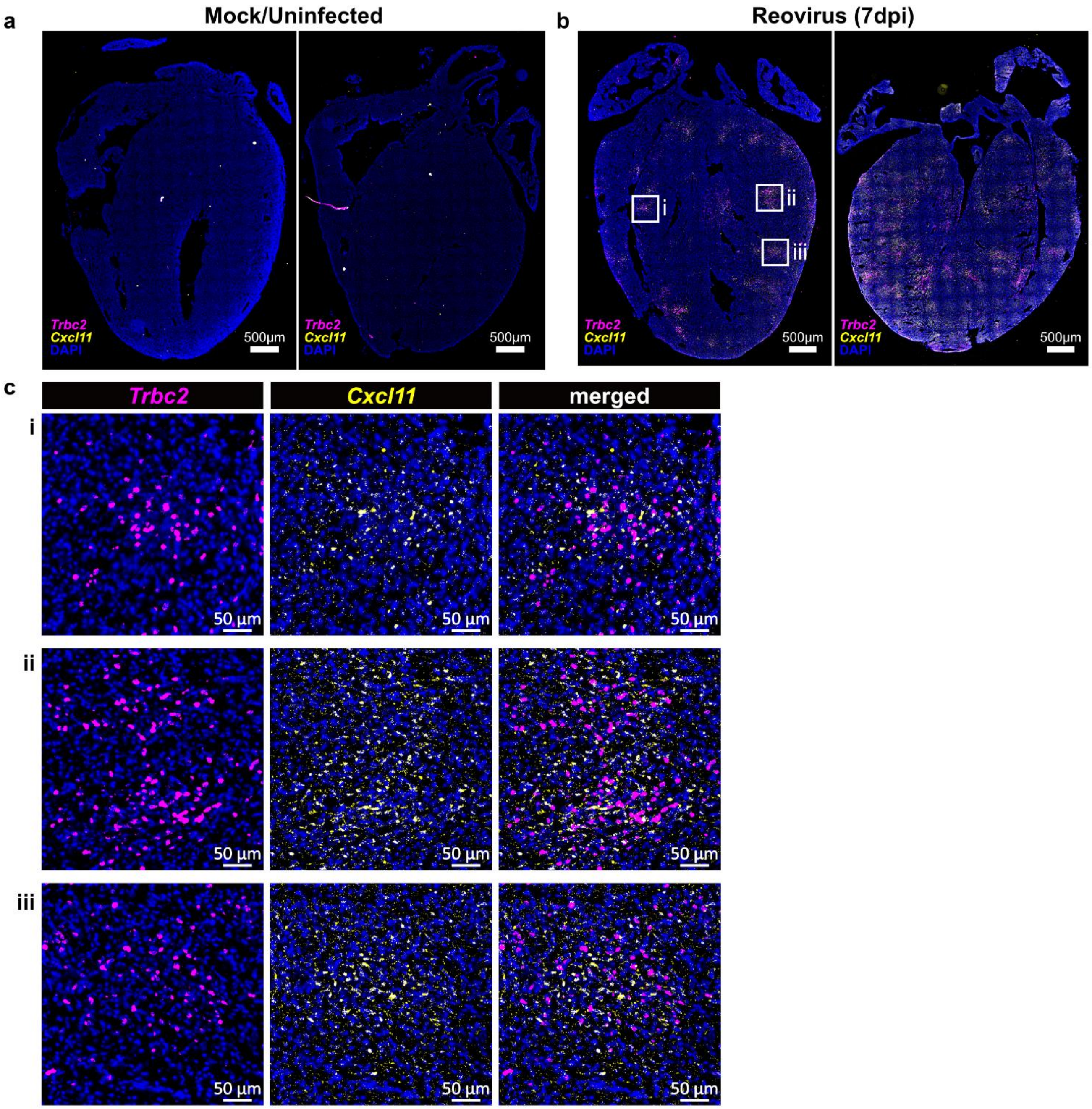

Figure S10. Single-molecule RNA fluorescence in situ hybridization identifies spatial colocalization of *Cxcl11* to T cell infiltration loci in reovirus-induced myocarditis. **(a)** FISH targeting the T cell marker *Trbc2* and the pseudogene *Cxcl11* in uninfected hearts and **(b)** infected hearts. Two technical replicates from the same heart sample are shown. Boxes in (b) mark myocarditic loci for which high resolution views are shown in **(c)**. Brightness and contrast of whole heart images was adjusted on entire images for visualization purpose.

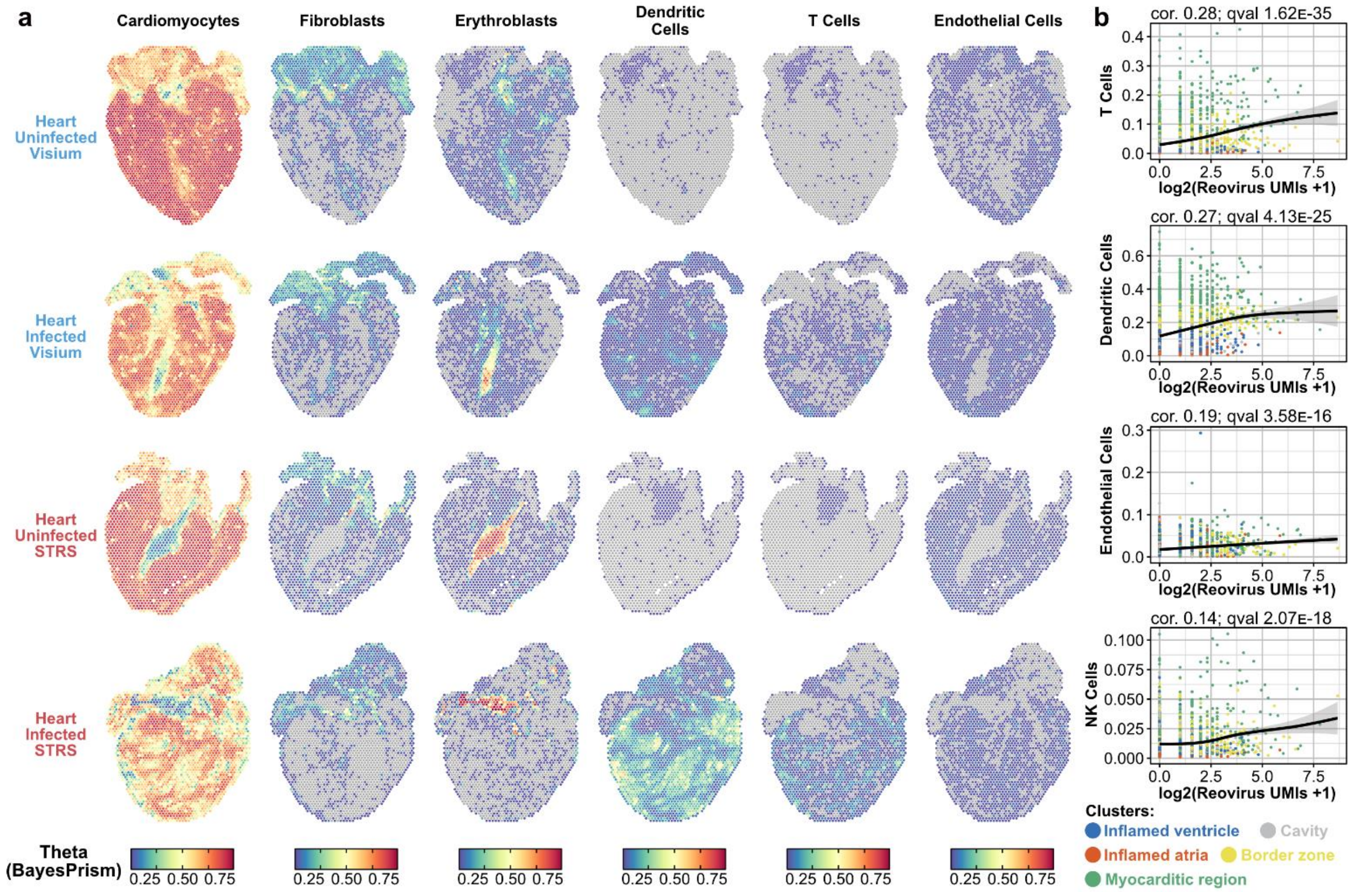

Figure S11. Spot deconvolution analysis of Visium and STRS data of mock and reovirus-infected heart samples. **(a)** Spatial maps of select cell types. The color scale shows the theta value, or fraction of reads attributed to each cell type, as computed by BayesPrism. **(b)** Correlation of pulldown-enriched reovirus UMIs and cell type deconvolution results. The line shows log2-normalized Reovirus counts (x-axis) and BayesPrism-derived theta values (y-axis) fit to a general additive model and error bands show a 95% confidence interval (Methods). Correlation and q-value reported are from general additive model analysis.

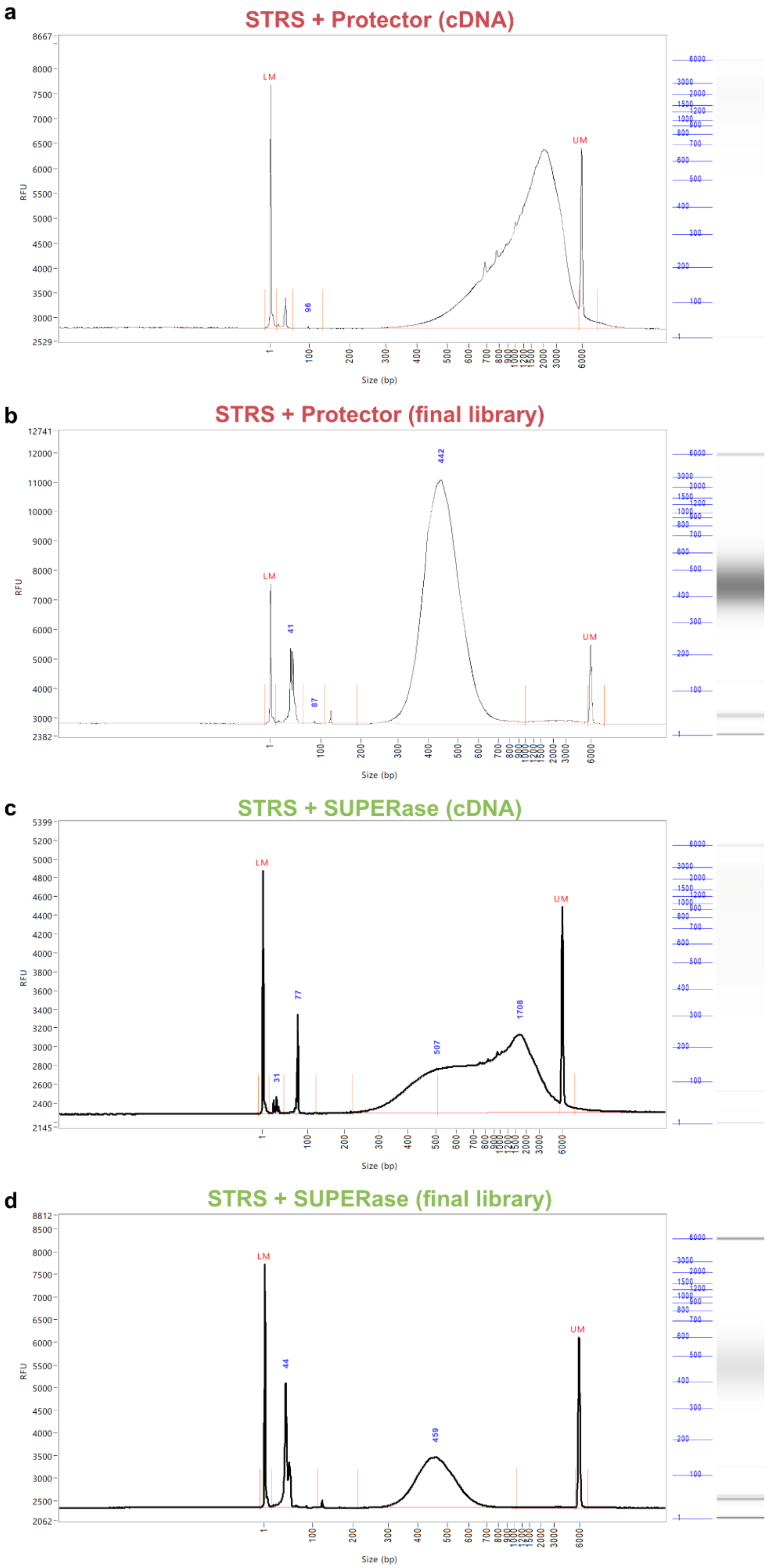

Figure S12. DNA fragment analysis of Spatial Total RNA-Sequencing (STRS) cDNA (pre-tagmentation) and final libraries (post-tagmentation). Data is shown for libraries generated using either Protector RNase Inhibitor (**a-b**) or SUPERase in RNase Inhibitor (**c-d**) during in situ polyadenylation (see **Methods**). Samples shown are STRS_3A (GSM6034864; STRS + Protector, Uninfected Heart) and STRS_2B (GSM6034862; STRS + SUPERase, Skeletal Muscle uninjured/D0B and 2dpi).
