# Supplementary material for: Spatial mapping of the total transcriptome by in situ polyadenylation
Source: Nat Biotechnol. 2022 Nov 3;41(4):513–20. doi: 10.1038/s41587-022-01517-6 (PMC10110464; doi:10.1038/s41587-022-01517-6)
Supplement: Supplementary file 2 — Reporting Summary [file 41587_2022_1517_MOESM2_ESM.pdf]

nature research

Corresponding author(s): NBT-RA56865

Last updated by author(s): Sep 7, 2022

Reporting Summary

Nature Research wishes to improve the reproducibility of the work that we publish. This form provides structure for consistency and transparency in reporting. For further information on Nature Research policies, see our Editorial Policies and the Editorial Policy Checklist.

Statistics

For all statistical analyses, confirm that the following items are present in the figure legend, table legend, main text, or Methods section.

n/a	Confirmed	
☐	☒	The exact sample size (*n*) for each experimental group/condition, given as a discrete number and unit of measurement
☐	☒	A statement on whether measurements were taken from distinct samples or whether the same sample was measured repeatedly
☐	☒	The statistical test(s) used AND whether they are one- or two-sided<br>*Only common tests should be described solely by name; describe more complex techniques in the Methods section.*
☐	☒	A description of all covariates tested
☐	☒	A description of any assumptions or corrections, such as tests of normality and adjustment for multiple comparisons
☐	☒	A full description of the statistical parameters including central tendency (e.g. means) or other basic estimates (e.g. regression coefficient) AND variation (e.g. standard deviation) or associated estimates of uncertainty (e.g. confidence intervals)
☐	☒	For null hypothesis testing, the test statistic (e.g. *F*, *t*, *r*) with confidence intervals, effect sizes, degrees of freedom and *P* value noted<br>*Give P values as exact values whenever suitable.*
☐	☒	For Bayesian analysis, information on the choice of priors and Markov chain Monte Carlo settings
☐	☒	For hierarchical and complex designs, identification of the appropriate level for tests and full reporting of outcomes
☐	☒	Estimates of effect sizes (e.g. Cohen's *d*, Pearson's *r*), indicating how they were calculated

Our web collection on statistics for biologists contains articles on many of the points above.

Software and code

Policy information about availability of computer code

Data collection

No software was used to collect data in this study.

Data analysis

A detailed protocol for performing STRS as well as custom analysis scripts for aligning and processing STRS data can be found at https://github.com/mckellardw/STRS.

Preprocessing and alignment of Spatial Total RNA-Sequencing, single-nucleus total RNA-sequencing, Smart-Seq-Total, and VASA-seq data
All code used to process and analyze these data can be found at https://github.com/mckellardw/STRS. An outline of the pipelines used for preprocessing and alignment is shown in Fig S1a.

Reads were first trimmed using cutadapt v3.4 to remove the following sequences: 1) poly(A) sequences from the three prime ends of reads, 2) the template switch oligonucleotide sequence from the five prime end of reads which are derived from the Visium Gene Expression kit (sequence: CCCATGTACTCTGCGTTGATACCACTGCTT), 3) poly(G) artifacts from the three prime ends of reads, which are produced by the Illumina two-color sequencing chemistry when cDNA molecules are shorter than the final read length, and 4) the reverse complement of the template switching oligonucleotide sequence from the five prime ends of reads (sequence: AAGCAGTGGTATCAACGCAGAGTACATGGG). Next, reads were aligned using either STAR v2.7.10a or kallisto v0.48.0. Workflows were written using Snakemake v6.1.0.

For STAR, the genomic reference was generated from the GRCm39 reference sequence using GENCODE M28 annotations. For STAR alignment, the following parameters, based on work by Isakova et al, were used: outFilterMismatchNoverLmax=0.05, outFilterMatchNmin=16, outFilterScoreMinOverLread=0, outFilterMatchNminOverLread=0, outFilterMultimapNmax=50. Aligned reads were deduplicated for visualization using umi-tools v1.1.2. Aligned and deduplicated reads were visualized with Integrated Genome Viewer v2.13.0. Normalized gene position plots and genomic loci profiles were generated using Qualimap v2.2.2.a.

For kallisto, a transcriptomic reference was also generated using the GRCm39 reference sequence and GENCODE M28 annotations. The

default k-mer length of 31 was used to generate the kallisto reference. Reads were pseudoaligned using the `kallisto bus` command with the chemistry set to "VISIUM" and the `fr-stranded` flag activated to enable strand-aware quantification. Pseudoaligned reads were then quantified using bustools v0.41.0. First, spot barcodes were corrected with `bustools correct` using the "Visium-v1" whitelist provided in the Space Ranger software from 10x Genomics. Next, the output bus file was sorted and counted using `bustools sort` and `bustools count`, respectively. To estimate the number of spliced and unspliced transcripts, reads pseudoaligned using kb-python v0.26.0, using the "lemanno" workflow.

Spots were manually selected based on the H&E images using Loupe Browser from 10x Genomics. Spatial locations for each spot were assigned using the Visium coordinates provided for each spot barcode by 10x Genomics in the Space Ranger software ("Visium-v1_coordinates.txt"). Downstream analyses with the output count matrices were then performed using Seurat v4.0.4. In addition to manual selection, spots containing fewer than 500 detected features or fewer than 1000 unique molecules were removed from the analysis. Counts from multimapping features were collapsed into a single feature to simplify quantification. Gene biotype percentages were computed according to gene biotypes provided in the GENCODE M28 annotations.

Single-nucleus data were preprocessed and aligned as described above, with a different barcode whitelist matching the 10x Genomics Chromium v3 chemistry. Count matrices were filtered for cells with more than 750 unique molecules and less than 5% of reads mapping to mitochondrial genes. Counts were then log-normalized with Seurat. Cells were merged and differential gene expression analysis was performed between the standard and in-situ-polyadenylated nuclei using the `FindMarkers()` function. A Wilcoxon ranked sum test was used for differential gene expression analysis.

Raw fastq files for VASA-drop7 samples were downloaded from GEO (GSE176588) using parallel-fastq-dump (v0.6.5). Reads were trimmed using cutadapt v3.4 to remove poly(A) and poly(G) sequences. Reads were then aligned and quantified using kallisto/BUStools as described above. The `--technology` flag for `kallisto bus` was set to '0,6,22:0,0,6:1,0,0' for cell barcode and UMI identification to reflect the modified fastq files authors uploaded to GEO. Gene counts from multimapping features were collapsed into a single feature.

Raw fastq files for Smart-Seq-Total samples were downloaded from GEO (GSE151334) using parallel-fastq-dump (v0.6.5). Reads were then pseudoaligned using `kallisto quant` with the `--fragment-length` flag set to 75 and the `--sd` flag set to 10. Transcript counts were converted to gene counts according to GENCODE M28 gene symbols, then counts from multimapping features were collapsed into a single feature.

Rarefaction analysis of Visium and Spatial Total RNA-Sequencing data
Raw fastq files for each library were randomly down-sampled four times using seqtk v1.2 to final read counts totaling between 100,000 and 50,000,000 reads. Final libraries were then pseudoaligned using the kallisto pipeline described above.

Annotation-free quantification of transcriptionally active regions in single-nucleus RNA-sequencing data
The `from_STARsolo` version of the TAR-scRNA-seq pipeline was used with the outputs from reads aligned with STAR for single-nucleus RNA-sequencing data. Default parameters were used for `MERGEBP` (500) and `THRESH` (10000000) for TAR merging and filtering, respectively. Count matrices generated by TAR-scRNA-seq were subset based on cell barcodes which remained after standard quality control.

- Mature microRNA quantification
For STRS data: after trimming (see above), barcode correction with STAR v2.7.10a, and UMI-aware deduplication with umi-tools v1.1.2, reads were split across all 4992 spot barcodes and analyzed using miRge3.0 v0.0.920. Reads were aligned to the miRbase reference provided by the miRge3.0 authors. MiRNA counts were log-normalized according to the total number of counts detected by kallisto and scaled using a scaling factor of 1000. For small RNAseq data: Reads were first trimmed using trim_galore v0.6.5. Reads were then aligned and counted using miRge3.0 v0.0.9.

- Unsupervised clustering and differential gene expression analysis of spot transcriptomes
Spot UMI counts as generated by kallisto were used. First, counts were log-normalized and scaled using default parameters with Seurat. Principal component analysis was then performed on the top 2000 most variable features for each tissue slice individually. Finally, unsupervised clustering was performed using the `FindClusters()` function from Seurat. The top principal components which accounted for 95% of variance within the data were used for clustering. For skeletal muscle samples, a clustering resolution was set to 0.8. For heart samples, clustering resolution was set to 1.0. Default options were used for all other parameters. Finally, clusters were merged according to similar gene expression patterns and based on histology of the tissue under each subcluster.

Differential gene expression analysis was performed using the `FindAllMarkers()` function from Seurat. Default parameters were used, including the use of the Wilcoxon ranked sum test to identify differentially expressed genes. To identify features enriched in the skeletal muscle STRS datasets, all Visium and STRS were first merged and compared according to the method used (Visium vs. STRS). To identify cluster-specific gene expression patterns, skeletal muscle samples were first clustered as described above individually. STRS samples were then merged, and differential gene expression analysis was performed across the three injury region groups.

- Cell type deconvolution of Visium and STRS datasets
Cell type deconvolution of skeletal muscle Visium and STRS data was performed as previously5 using BayesPrism (previously known as "Tumor microEnvironment Deconvolution", TED, v1.0; github.com/Danko-Lab/TED). We used the "scMuscle" dataset generated in McKellar et al as a single-cell transcriptomic reference for skeletal muscle. For heart samples, we used all mock and infected single-cell RNA-sequencing samples generated by Mantri et al. Highly and differentially expressed genes across cell types were identified with differential gene expression analysis using Seurat (FindAllMarkers, using Wilcoxon Rank Sum Test). The resulting genes were filtered based on average log2-fold change (avg_logFC > 1) and the percentage of cells within the cluster which express each gene (pct.expressed > 0.5), yielding ~1000 genes in both single-cell references. Mitochondrial and ribosomal protein genes were removed from this list, in line with recommendations from the BayesPrism authors. For each of the cell types, mean raw counts were calculated across the ~1,000 genes to generate a gene expression profile for BayesPrism. Raw counts for each spot were then passed to the run.Ted function, using the "GEP" option for input.type and default parameters for the remaining inputs. Final Gibbs theta values were used as estimates for the fraction of transcripts from each spot that were derived from each of the cell types. In plots (Fig S8, S11), a minimum threshold value for theta of 0.01 was used. For skeletal muscle, after deconvolution all spots were merged. Principal component analysis was performed on the non-thresholded BayesPrism theta values using Seurat.

For manuscripts utilizing custom algorithms or software that are central to the research but not yet described in published literature, software must be made available to editors and reviewers. We strongly encourage code deposition in a community repository (e.g. GitHub). See the Nature Research guidelines for submitting code & software for further information.

Data

Policy information about availability of data

All manuscripts must include a data availability statement. This statement should provide the following information, where applicable:

- Accession codes, unique identifiers, or web links for publicly available datasets
- A list of figures that have associated raw data
- A description of any restrictions on data availability

Previously published spatial RNA-sequencing data were downloaded from Gene Expression Omnibus (GEO) and are available under the following accession numbers; regenerating skeletal muscle5 GSE161318, infected heart tissue GSE189636. Spatial Total RNA-Sequencing data generated in this study can be found on GEO under the accession number GSE200481. Small RNA-sequencing data are available on GEO under the accession number GSE200480. Single-nucleus RNA-sequencing data for C2C12 nuclei with and without in situ polyadenylation can be found on GEO under the accession number GSE209780. Public datasets for Smart-Seq-Total (GSE151334), VASA-seq (GSE176588), the small RNA-sequencing atlas (GSE119661), and the viral myocarditis single-cell RNA-sequencing reference (GSE189636) were downloaded from GEO. The skeletal muscle single-cell RNA-sequencing reference was downloaded as a Seurat object from Dryad (https://datadryad.org/stash/dataset/doi:10.5061%2Fdryad.t4b8gtj34). A detailed protocol for performing STRS as well as custom analysis scripts for aligning and processing STRS data can be found at https://github.com/mckellardw/STRS.

Field-specific reporting

Please select the one below that is the best fit for your research. If you are not sure, read the appropriate sections before making your selection.

☒ Life sciences ☐ Behavioural & social sciences ☐ Ecological, evolutionary & environmental sciences

For a reference copy of the document with all sections, see nature.com/documents/nr-reporting-summary-flat.pdf

Life sciences study design

All studies must disclose on these points even when the disclosure is negative.

Sample size	One sample per condition was collected using the protocol in this manuscript, but six total samples were collected and compared to ensure the fidelity of the protocol developed.
Data exclusions	Data collected using a previous variant of our method which used an ineffective RNase inhibitor were excluded from these analyses, but are publicly available on GEO.
Replication	We processed six samples using the method described in this manuscript, and assessed quality of each sample to ensure the repeatability of Spatial Total RNA-Sequencing.
Randomization	Mice, from which injured hindlimb muscles were collected, were injured and collected so that multiple time points were collected at once (up to four samples in a single day), to reduce experimental bias. Mice were randomly selected for each injury time point. Mice were also randomly selected for viral infection experiments. Final heart samples used to assess viral infection were selected based on infection phenotype as seen in H&E imaging.
Blinding	During the development of the method described in this study, blinding was not necessary.

Reporting for specific materials, systems and methods

We require information from authors about some types of materials, experimental systems and methods used in many studies. Here, indicate whether each material, system or method listed is relevant to your study. If you are not sure if a list item applies to your research, read the appropriate section before selecting a response.

Materials & experimental systems

n/a	Involved in the study
☒	☐ Antibodies
☒	☐ Eukaryotic cell lines
☒	☐ Palaeontology and archaeology
☐	☒ Animals and other organisms
☒	☐ Human research participants
☒	☐ Clinical data
☒	☐ Dual use research of concern

Methods

n/a	Involved in the study
☒	☐ ChIP-seq
☒	☐ Flow cytometry
☒	☐ MRI-based neuroimaging

Animals and other organisms

Policy information about studies involving animals; ARRIVE guidelines recommended for reporting animal research

Laboratory animals	For skeletal muscle samples, adult female C57BL/6J mice were obtained from Jackson Laboratories (#000664; Bar Harbor, ME) and were used at 6 months of age. For heart samples, confirmed pregnant female C57BL/6J mice were ordered from Jackson Laboratories to be delivered at embryonic stage E14.5.
Wild animals	No wild animals were used in this study.
Field-collected samples	No field-collected samples were used in this study.
Ethics oversight	The Cornell University Institutional Animal Care and Use Committee (IACUC) approved all animal protocols, and experiments were performed in compliance with its institutional guidelines.

Note that full information on the approval of the study protocol must also be provided in the manuscript.
